# A lactobacilli-based probiotic but not its postbiotic reduces intestinal inflammatory pathways expression in broilers fed a non-starch polysaccharide rich challenge diet^☆^

**DOI:** 10.1016/j.psj.2025.106159

**Published:** 2025-11-26

**Authors:** Samuel C.G. Jansseune, Wouter H. Hendriks, Fany Blanc, Jordi Estellé, Nicolas Bruneau, Jean-Luc Coville, Marie-Hélène Pinard-van der Laan

**Affiliations:** a Animal Nutrition Group, Department of Animal Sciences, Wageningen University & Research, Wageningen, the Netherlands; b Université Paris‐Saclay, INRAE, AgroParisTech, GABI, Jouy‐en‐Josas, France; c Idena, Sautron, France

**Keywords:** Inflammation, Transcriptome, Probiotic, Postbiotic, Lactobacilli

## Abstract

Diets rich in non-starch polysaccharides (**NSP**) are known to challenge broiler growth and induce gut inflammation. Here, a lactobacilli-based probiotic (**Pro**) and its derived postbiotic (**Post**) were investigated when supplemented to an NSP-rich challenge diet (**Ctrl**) fed to broilers reared under commercial conditions. Treatments had 8 pen replicates with 40 broilers each. On day 35, for 3 broilers/pen, the microbiota, volatile fatty acids and semi-polar metabolome composition were determined in ileal and caecal digesta. In addition, changes in the transcriptome of jejunal, ileal and caecal tonsil tissues as well as in the blood were investigated by RNA-seq. Apparent ileal nutrient digestibility and tibia health were also assessed. The Pro affected the relative abundance of four operational taxonomic units (**OTU**) in ileal and none in caecal digesta. The Post affected the relative abundance of nine OTU in ileal and two in caecal digesta. Microbiota diversity indexes and the semi-polar metabolome were not affected by Pro and Post. According to gene set enrichment analysis of Gene Ontology (**GO**) and the Kyoto Encyclopedia of Genes and Genomes (**KEGG**) pathways, the jejunal tissue transcriptome of Pro *v.s.* Ctrl birds had upregulated pathways linked to cell activity, energy production and DNA transcription. In ileal tissue, similar pathways had a significant enrichment score. In jejunal tissue, downregulated pathways with Pro were linked to immunity and inflammation. In caecal tonsil tissue, Pro was associated with a negative enrichment score of immune and detoxification-related pathways in KEGG and GO. The Pro had minor effects on the blood transcriptome. Compared to Ctrl, Post had minor effects on the jejunal, ileal and caecal tonsil tissues transcriptome, and no effects on the blood transcriptome. Overall, Pro and Post elicited different physiological effects with Pro but not Post supplementation inducing a clear pattern of potential mechanisms. Results indicate that the lactobacilli-based probiotic induced beneficial effects by lowering inflammation in the gut, and that these effects likely originate from the more proximal intestine.

## Introduction

Ingredients for poultry feed are becoming scarcer and more expensive (Singh and Kim, 2021) facilitating the dietary inclusion of alternative ingredients. Due to the presence of anti-nutritional compounds, *e.g*. non-starch polysaccharides (**NSP**), the use of these alternative ingredients can result in impaired gut health and reduced welfare of the birds with subsequent effects on growth performance (Beski et al., 2015; Polovinski-Horvatović, 2021; Lannuzel et al., 2022). Continued efforts have focussed on finding in-feed components or formulations to promote broiler gut health. Among such additives are pro- and postbiotics which have been reported to have the potential to promote gut health in broilers (Humam et al., 2019; Jha et al., 2020).

The beneficial effects of pro- and postbiotics on broiler gut health have been reported to be effectuated through direct interaction with immune and intestinal epithelial cells, and indirectly through modulation of the gut microbiota and fermentation (Fathima et al., 2022). The caecal tonsils, in this respect, play a key role in chicken immunity and can be affected by changes in microbiota composition (Berndt et al., 2007; Setta et al., 2012; Cazals et al., 2022) and through the use of in-feed probiotics (Haghighi et al., 2008; Hu et al., 2015). In their meta-analysis, Yosi and Metzler-Zebeli (2023) also reported that certain probiotics can affect broiler jejunal and ileal tissue gene expression related to gut barrier functions and immunity under non-challenging or challenged conditions, with possible beneficial effects on gut integrity. In other species, lactobacilli pro- or postbiotics have been reported to reduce gut inflammation in a mice inflammation model (Martín et al., 2019; Monteros et al., 2021; Wang et al., 2024), ex vivo explants from humans (Compare et al., 2017), and can modulate the peripheral blood transcriptome (Burton et al., 2018). Probiotics and postbiotics have been repeatedly studied for their effects on the broiler gut microbiota, which can include modifications of its richness, diversity, composition or activity (Li et al., 2017b; Rodrigues et al., 2020; Danladi et al., 2022a; Chai et al., 2023; Yang et al., 2023). The gut microbiota impact gut health, interact with immune cells, can modulate gut morphological structure and can consequently influence nutrient digestion and absorption (Diaz Carrasco et al., 2019; Liao et al., 2020; Fathima et al., 2022; Kogut, 2022). The gut microbiota participate in shaping the gut metabolome. In poultry, the most studied microbial metabolites are the short-chain fatty acids (SCFA) (Liu et al., 2021), which can be affected by Pro and Post (Jansseune et al., 2024a). In contrast to SCFA analysis, (un)targeted metabolomics, a recently developed discipline, provides a tool to determine in great detail a large number of low-molecular-weight microbial and cellular metabolites (< 1000 Da) (Gertsman and Barshop, 2018), representing a promising tool to investigate pro- or postbiotic effects in poultry (Wu et al., 2022; Jansseune et al., 2024a).

The presence of a challenge appears to be essential for the observation of pro- and postbiotics effects (Fuller, 1986; Otutumi et al., 2012; Jansseune et al., 2024c). Challenging conditions for broilers can be, amongst others, dietary or stress associated (Akinyemi and Adewole, 2021; Wang et al., 2023b). When levels of dietary nutrients are insufficient (*e.g*. protein, amino acids), probiotics have been reported to have a beneficial effect on growth and feed conversion (Mikulec et al., 1999; Imari et al., 2023) while when diets contain all the nutrients in sufficient levels, improvements by probiotics are absent (Mikulec et al., 1999; Jansseune et al., 2024c). Diets rich in NSP are known to challenge broiler growth (Lazaro et al., 2003; Mehrabadi and Jamshidi, 2019; Jansseune et al., 2024b, c) and to induce inflammation in the gut (van Krimpen et al., 2017; Cardoso Dal Pont et al., 2021). Recently, beneficial effects of a pro- and postbiotic additive were reported when an NSP rich diet was fed as a challenge to broilers (Jansseune et al., 2024c) although this effect could not be reproduced (Jansseune et al., 2024b).

It has been reported (Jansseune et al., 2025) that at d35, a lactobacilli-based probiotic (**Pro**) and its derived postbiotic (**Post**) affected growth performance parameters in broilers, when supplemented to an NSP-rich challenge diet. The current study aimed to obtain a clearer understanding of the mode(s) of action of Pro and Post when supplemented to an NSP-rich challenge diet (**Ctrl**) on broiler metabolism and physiology. Ileal and caecal digesta were investigated for their microbiota, SCFA and semi-polar metabolome composition. Changes in the transcriptome of jejunal, ileal and caecal tonsil tissues as well as in the blood were investigated by RNA-seq in Ctrl, Pro and Post fed birds. Apparent ileal digestibility and leg health were determined as parameters potentially affected by pro- or postbiotics.

## Materials and methods

### Ethic statement

The experiment was approved by the French Ministry of Education, Higher Education and Research (Ministère de l'Éducation nationale, de l'Enseignement supérieur et de la Recherche) under the protocol No. APAFIS #44135-2023071114126771 v5, and carried out according to the French legislation.

### Birds housing and management

A large batch of one-day-old male Ross 308 broilers originating from 50-week-old broiler breeders was purchased from a commercial hatchery (Couvoir de Cleden, Cleden Poher, France), with 960 chicks selected, based on individual weights and distributed across 24 pens with 40 broilers each, so that all pens had a similar average chick body weight (**BW**) (∼44.9 ± 2.42 g) and distribution. Each experimental group had 8 pen replicates. Pens were 1.90 × 1.25 × 0.8 m (*L* × *W* × *H*) with wood shavings as floor covering. They were located along the wall of air entries on one side of a commercial, 1200 m² Colorado-type building. Water and feed per pen were provided *ad libitum* through 5-6 nipple drinkers and one 40 cm diameter Hung pan feeder (Josse, Montauban de Bretagne, France). The photoperiod was 24 h light until d4 and then 20 h from d5 to 37. Ambient temperature started at 32 °C on d1 and, thereafter, gradually reduced linearly to 23 °C on d22. Birds were inspected daily for lethargy, prostration and lameness, and culled if found to be unhealthy. The total duration of the experiment was 37 days (d1 to 37).

As in normal practice, at day of hatch, broilers were spray vaccinated against infectious bronchitis virus (**IBV**) (Nobilis BI H120, Nobilis, MSD santé animale, Beaucousé, France) and coccidiosis (Paracox-5, Intervet UK Ltd, United-Kingdom), and were vaccinated through drinking water against infectious bursal disease virus (**IBDV**) (HIPRAGUMBORO G97, Laboratorios Hipra, Amer, Spain) on d17.

### Experimental treatments

A randomised complete block design containing three treatment groups (Ctrl, Pro and Post) was used. A pelleted diet was formulated based on commercial standards for nutrients levels for Ross 308 broilers and provided adequate levels of all nutrients to the birds (Table 1). The Ctrl diet was supplemented with Pro or Post obtained from STI biotechnologie (Maen Roch, France). The Pro is the biomass resulting from the co-fermentation of a milk-based substrate by a mixture of L. rhamnosus CNCM-I-3698 and L. formosensis CNCM-I-3699, and Post is derived from heat-inactivated (high temperature short time) biomass of Pro. Both Pro and Post biomasses originated from a mixture of four production batches containing 3 × 10^9^ colony-forming units per g of biomass after fermentation and were dried as powder on corn flour. Corn flour was used for the Ctrl in equal amounts as provided by Pro and Post to account for the presence of corn flour as carrier in the latter two. The Pro and Post biomasses were included in the diet at 50 and 500 g biomass/t as is, respectively, from d1 to 14, and at 40 and 400 g biomass/t as is, respectively, from d14 to 37. From d35 to 37, the finisher diet contained 1.5 g TiO_2_/t feed as is for a digestibility study.

**Table 1:** Ingredient and calculated composition including energy content of the standard and challenge starter (0-14 d), grower (14-28 d) and finisher (28-37 d) diet for broilers.

Composition	Starter	Grower	Finisher
**Ingredient (% as is)**			
Corn	13.982	12.509	13.000
Wheat	28.000	30.000	29.949
Barley	10.000	10.000	10.000
Rye	7.500	12.500	18.000
Soybean meal	32.000	26.800	20.700
Limestone	1.640	1.110	0.850
Mono calcium phosphate dihydrate	1.400	0.860	0.800
Sodium chloride 99 %	0.280	0.250	0.260
Sodium bicarbonate	0.100	0.110	0.130
Soy oil	3.890	4.650	4.890
DL-methionine 99 %	0.301	0.274	0.231
L-Lysine HCL 98 %	0.242	0.257	0.329
L-Threonine 98 %	0.134	0.129	0.163
L-Valine 96.5 %	0.031	0.024	0.051
L-Isoleucine 90 %	-	0.027	0.067
L-Arginine 98 %	-	-	0.080
Premix^1^	0.500	0.500	0.500
**Calculated (% as is)**			
Dry matter	88.8	88.9	88.8
Crude protein	21.5	19.6	17.5
Crude fat	5.28	6.02	6.28
Starch	36.9	40.0	43.3
Ash	6.61	5.26	4.65
Fibre^2^	13.9	13.7	13.3
Total non-starch polysaccharides^2^	12.4	12.2	11.8
Soluble non-starch polysaccharides^2^	3.05	2.98	2.85
Cellulose	2.86	2.76	2.63
Dig. Methionine	0.59	0.54	0.47
Dig. Methionine+cystine	0.93	0.86	0.75
Dig. Lysine	1.32	1.20	1.10
Dig. Threonine	1.32	1.20	1.10
Dig. Valine	0.90	0.82	0.75
Dig. Arginine	1.29	1.14	1.05
Calcium	0.98	0.67	0.55
Available phosphorous	0.48	0.35	0.33
Chlorine total	0.27	0.25	0.27
Sodium total	0.15	0.14	0.15
Apparent metabolizable energy (MJ/kg as is)	12.13	12.72	12.88

1 Supplied per kg premix: 2,000,000 IU retinyl acetate, 500,000 IU cholecalciferol, 10 g DL-α-tocopherol, 460 mg menadione, 400 mg thiamine, 1,500 mg riboflavin, 700 mg pyridoxine-HCL, 4 mg cyanocobalamin, 7 g niacin, 2.4 g D-pantothenic acid, 92 g choline chloride, 200 mg folic acid, 40 mg biotin, 53 g FeSO_4_·H_2_O, 9.6 g CuSO_4_·5H_2_O, 28 g MnO, 33 g ZnSO_4_·H_2_O, 360 mg KI, 112 mg Na_2_SeO_3_.

2 Values were calculated from levels reported by Knudsen (2014) and restricted to cereals and soybean meal.

### Samples collection

On d35, three broilers per pen were randomly selected for sampling, for a total of 24 broilers per treatment group. First, blood was collected from the wing vein in EDTA-tubes (BD Vacutainer®, Plymouth, England) and, immediately after collection, 200 µL of blood was diluted in phosphate-buffered saline (**PBS**) v/v and mixed with 2 mL of TRIzol lysis reagent (Invitrogen, Waltham, USA) and immediately frozen in liquid nitrogen before being stored at −80 °C. Following blood sampling, birds were euthanized by electronarcosis followed by cervical dislocation. The abdominal cavity was opened, and the gut segment middle jejunum, middle ileum and caeca were identified. The digesta from 3 cm left or right of the middle of the segment were obtained by gentle squeezing of the segments by hand for microbiota and metabolome analysis. Tissue samples from the middle of the jejunum and ileum as well as the caecal tonsils were collected and washed in PBS 1X. All blood, tissue, and digesta samples were immediately frozen in liquid nitrogen and stored at −80 °C.

For tibia measurements, soft tissues of both legs were manually removed from the tibia using a scalpel. The proximal end of both tibias was longitudinally cut with a scalpel to visually grade tibia dyschondroplasia (**TD**) score (0: absence, 1: intermediate, 2: severe), and to measure the width of the articular cartilage, proliferation zone and mineralisation zone with a digital calliper.

For apparent ileal digestibility, 10 broilers per pen were selected during the weighing at d35. First, the average pen weight was determined by collective weighing of all birds per pen. Then randomly selected birds were individually weighed and the first five with a weight within 10 % below and five within 10 % above the mean were returned to the same pen and fed the finisher diet containing TiO_2_. On d37, the 10 birds per pen were euthanised by euthasol injection (Dechra, Northwich, England) in the occipital sinus, weighed and the total ileum (from Meckel’s diverticulum to the ileocaecal valve) was isolated using surgical soft clamps. The ileal contents of the birds were flushed from the intestinal segment with 50 mL demineralised water, pooled per pen and frozen on dry ice prior to being stored at −20 °C. On d37, pen feed intake was also recorded and feed conversion ratios per pen calculated.

### Nutrient digestibility assessment

Feed and freeze-dried ileal digesta samples were ground to pass a 1 mm sieve and then analysed in duplicate for dry matter (method ISO 6496 (1999)), ash (method ISO 5984 (2003)), N (method ISO 16634–1 (2008)), Ca and P (method ISO 16943 (2017)) contents. Non-starch polysaccharides were analysed using the procedure described by Englyst et al. (1994) and detailed by Garçon et al. (2023). Titanium content in feed and ileal digesta was determined as presented by Schaafstra et al. (2018).

### Microbiota composition analysis

For each digesta sample, DNA from 200 mg defrosted digesta was extracted following the standardized protocol described by Borey et al. (2020). Then, DNA quantity and quality was determined prior to DNA amplification and sequencing of the V3-V4 hyper-variable region of the 16S rRNA coding gene by the INRAE@BRIDGe platform, as described by Jansseune et al. (2024a). Following the sequencing, identification of swarm clustered operational taxonomic units (OTU) was performed with the FROGS [Find Rapidly OTUs with Galaxy Solution, (Escudié et al., 2018)] pipeline on Galaxy (v.4.1.0) (Afgan et al., 2018). The merging, denoising and dereplication of the sequenced reads were performed with a mismatch rate < 0.1 and sequence length of 433 to 460. Following identification of OTUs and removal of chimera, the filtering was performed with a minimum relative abundance threshold of 0.005 %, a blast coverage > 0.99, an identity > 0.95 and a presence in at least five of all samples. No compositional analyses were performed on the jejunal microbiota as insufficient DNA could be recovered.

### Metabolome analysis

***Volatile Short-Chain Fatty Acid Analysis*.** Acetate, propionate, butyrate, isobutyrate, valerate, isovalerate) and (iso-)caproate concentration in defrosted ileal and caecal digesta samples were determined by gas chromatography as reported by Bernard et al. (2024). Briefly, between 100 and 250 mg of defrosted digesta were diluted 2 times w/v of deionised water. Samples were homogenised and mixed for 2 h at 4 °C before centrifugation (12,000 × g; 15 min; 4 °C). The supernatant was collected and weighed, and 10 % v/v of phosphotungstic acid saturated solution (Sigma-Aldrich, Saint-Louis, USA) was added for protein precipitation overnight at 4 °C. As an internal standard, 10 µL of 2-ethylbutyrate (Sigma-Aldrich) was added to 40 µL of acidified supernatant, and the solution was analysed using a gas–liquid chromatograph (GC-FID 7890B, Agilent, Santa Clara, USA) and a fused silica capillary column Supelco (n°25326, Sigma-Aldrich). All samples were analysed in duplicate. Data were collected and peaks were integrated using Agilent OpenLab Chemstation software (v.2.3.53).

***Semi-Polar Metabolome Analysis*.** An accurately weighed amount (50-100 mg) of defrosted ileal or caecal digesta was mixed with 4 times w/v ultra-pure water and homogenized by shaking using a bead beater (Star beater, VWR, Radnor, USA) at 30 Hz for 4 min, using 1.4 mm ceramic beads (n°19-645, Revvity, Waltham, USA). Each homogenized sample was centrifuged (16,000 × g, 10 min, 4 °C, CENT 5430, Eppendorf, Hamburg, Germany) prior to supernatant collection. The latter was centrifuged (15,000 × g, 5 min, 4 °C, CENT 5430) through a 0.22 µm cellulose acetate filter (Corning, Corning, USA). A quality control sample was prepared by pooling equal aliquots from each ileal or caecal sample which was analysed every 6 samples. Untargeted metabolomics was performed for identification and relative quantification of the water soluble semi-polar metabolome as described by Iribarren et al. (2021). Metabolites in ileal and caecal digesta were analysed by MS-Omics (Vedbaek, Denmark). Compound identification was performed at four different levels (1, 2a, 2b, and 3) with identification criteria for 1) retention times (compared against in-house authentic standards), accurate mass (with an accepted deviation of 3 ppm), and MS/MS spectra, 2a) retention times (compared against in-house authentic standards) and accurate mass (with an accepted deviation of 3 ppm), 2b) accurate mass (with an accepted deviation of 3 ppm) and MS/MS spectra, and 3) accurate mass alone (with an accepted deviation of 3 ppm) with the libraries Human metabolome database and CHEBI. Features absent from more than 10 samples in all treatments, absent in more than 5 samples per treatment or with a coefficient of variation above 20 % over the quality control samples were excluded. Missing values were imputed with half of the minimal value of the feature and peak area data were log-transformed and Pareto-scaled.

### Transcriptome analysis

Tissue RNA was extracted using the NucleoSpin RNA kit (Macherey-Nagel, Düren, Germany) following the manufacturer’s instructions. Blood RNA was extracted as follows: 1 mL of frozen PBS and TRIzol-diluted blood sample was mixed with 2 mL TRIzol lysis reagent and incubated at room temperature for 12 min. Chloroform (600 µL) was added and the resulting mixture was incubated at room temperature for 7 min before centrifugation (18,000 × g, 15 min, 4 °C). The aqueous phase was collected, mixed with 900 µL of isopropyl alcohol and kept at −20 °C overnight. After centrifugation (18,000 × g, 40 min, 4 °C), the supernatant was removed. The pellet was washed with 75 % ethanol, dried, and suspended in 60 µL RNAse-free water by heating to 50 °C for 10 min. The samples were further submitted to a DNAse treatment (Thermo Fisher Scientific, Waltham, USA) by adding 10 µL of 10X buffer, 2.5 µL of RNAse Out, 3.5 µL of Turbo DNAse and 24 µL of diethylpyrocarbonate-treated water and heating the resulting solutions at 37 °C for 1 h. RNAse-free water (100 µL) and 200 µL phenol-chloroform-isoamyl alcohol (Sigma-Aldrich) was added and centrifuged (18,000 × g, 5 min, 4 °C). The supernatant was collected, mixed with 0.8 µL glycogen (Invitrogen), 1/10 volume of 3 M sodium acetate, 2.5 volumes of cold absolute ethanol and incubated overnight at −20 °C. After centrifugation (18,000 × g, 40 min, 4 °C), the supernatant was removed, the pellet washed with 75 % ethanol, dried and suspended in RNAse-free water heated at 50 °C for 10 min.

The quality of the total RNA was verified using a 2200 TapeStation RNA Screen Tape device (Agilent) and its concentration ascertained using an ND-1000 spectrophotometer (NanoDrop, Wilmington, USA). Libraries were prepared from 250 ng of total RNA with the Illumina TruSeq stranded total mRNA kit and were run on the 25B flow cell using the NovaSeq X Series 25B Reagent Kit (300 cycles) (Illumina) to obtain an average of 80 million reads per sample. After quality control of the raw reads with FastQC, a pseudo-alignment strategy to quantify the gene expression levels was applied using the kallisto v0.51.0 pseudoaligner (Bray et al., 2016) with the Chicken genome annotation available at Ensembl’s 106 release (https://ftp.ensembl.org/pub/release-113/fasta/gallus_gallus/cdna/) to obtain the estimated transcript abundances. Gene-level abundances were subsequently obtained using the tximport v.1.26.1 R package (Soneson et al., 2015) and used for differential abundance analyses as described below.

### Statistical analyses

All statistical analyses were performed with R v.4.0.3 (R Core Team, 2023). Probability or adjusted-p values < 0.05 and 0.05 ≤ *p* < 0.10 were considered significant and a trend, respectively for Pro and Post effects compared to Ctrl.

Treatment effects on means were analysed per contrast of interest (Pro *v.s*. Ctrl and Post *v.s*. Ctrl), using the general linear model procedure and type 2 ANOVA, while their effects on TD were analysed by ordinal logistic regression with the cumulative linked mixed model function for logistic regression (library lme4 v.1.1.31). Models included a fixed treatment effect and a random block factor. For tibia head growth zones, the grade of TD, the broiler BW and the leg location (right *v.s*. left) were included as additional co-variates. The latter two parameters were included similarly for the analysis of TD. Models were tested for residual normality by Shapiro test and, a log or a boxcox transformation was applied if normality was absent. If residual normality could not be reached with these two data transformation procedures, a rank analysis was performed. Equal variances between treatment groups was confirmed with the Levene test.

The α- (Observed, Chao1, Ace, Shannon, Simpson, inverse Simpson and Fisher) and β-diversity (Bray, Jaccard, Unifrac and Weighed Unifrac) indexes were determined with the Phyloseq package (v.1.42.0) and treatment effects for contrasts Pro *v.s*. Ctrl and Post *v.s*. Ctrl identified by ANOVA and PERMANOVA (vegan v.2.6.4), respectively, on the rarefied data, obtained with the rarefy_even_depth function (Phyloseq) and a seed. Models included a fixed treatment effect and a random block factor. PERMANOVA was performed with 9,999 permutations.

For the untargeted-metabolomic data, probability values of the ANOVA were corrected for multiple testing by Benjamini-Hochberg correction, and fold changes were calculated based on non-transformed peak area data. An orthogonal partial least square-discrimination analyses (OPLS-DA) were performed with the ropls package (v.1.4.2) (Thévenot et al., 2015) and 1,000 permutations to assess the overall effect of Pro and Post on the ileal and caecal metabolome. The goodness-of-fit and the predictive ability were represented by R2Y and Q2Y, respectively (Thévenot et al., 2015).

Differential abundance analyses of OTUs and gene mRNA between treatments were performed with edgeR (v.3.42.4) (Robinson et al., 2010) using the blocks as a cofactor. First, OTUs and gene mRNA were filtered for low presence in the samples. The OTUs present in less than 10 % of the samples per segment were removed from analysis. For gene mRNA, the ones detected in less than 20 % of the samples of a treatment, independently of the tissue, were excluded of the analysis. Normalisation was performed with normLibSizes. The identification of differentially abundant OTUs and differentially expressed genes (**DEG**) was performed using a genewise negative binomial generalized linear models with quasi-likelihood tests.

In addition, for transcriptome analyses a multidimensional scaling was performed with the plotMDS function from package limma, v.3.56.2 (Ritchie et al., 2015) and all genes. Then, a gene set enrichment analysis (**GSEA)** of Gene Ontology (**GO**) and Kyoto Encyclopedia of Genes and Genomes (**KEGG**) pathways using the function gseGO and gseKEGG from package clusterProfiler (v.4.8.3) (Wu et al., 2021) with default parameters and a seed. For treatments with DEG in a segment, an over-representation analysis of KEGG pathways was performed on up- and downregulated DEG separately with the function enrichKEGG (clusterProfiler). The gene list used for GSEA and over-representation analyses were ordered by fold changes and using the Entrez gene ID. The latter IDs were converted from the Ensemble gene ID with mapIds. In case of duplicated Entrez gene ID, the one with the highest absolute fold change was kept.

## Results

### Effects of pro and post on tibia dyschondroplasia and head growth zones

Table 2 presents the treatment effects on tibia parameters taking into account cofactors (leg location, BW and TD score) when applicable. The TD score was not affected by Pro but tended to be increased by Post (+31.6 %; *p* = 0.083) (Table 2). The Pro increased the articular cartilage width (+6.8 %) and relative width (+5.9 %) while decreasing the relative mineralization zone width (−4.4 %). The Post increased the articular cartilage width (+4.2 %) but did not affect its relative width. Post-B also increased the growth plate width (+1.9 %) and its relative width (+6.1 %) while decreasing the relative mineralisation zone width (−5.0 %). Interestingly, the side of the broiler leg affected the cartilage width (LSmean: right leg 3.25 mm, left leg 3.14 mm; *p* = 0.036) and tended to affect its relative width (LSmean: right leg 31.8 %, left leg 30.8 %; *p* = 0.055), but not the other parameters.

**Table 2:** Tibia dyschondroplasia score grading and least square mean tibia head growth zone of 35-days-old male Ross 308 broilers fed a control (Ctrl) diet supplemented with either a lactobacilli-based probiotic (Pro) or its derived postbiotic (Post) from day 1 onward.

Parameter	Ctrl	Pro	Post		Pooled SEM		p-value^2^	
							Pro	Post
Tibia dyschondroplasia grade^1^								
0	41	41	32		-		0.761	0.083
1	1	5	10		-			
2	0	2	3		-			
3	6	0	3		-			
Average	0.396	0.188	0.521		0.205			
Tibia head growth zone								
Width (mm)								
Articular cartilage	3.08	3.29	3.21		0.108		0.001	0.035
Growth plate	4.13	4.13	4.21		0.194		0.798	0.001
Mineralisation zone	3.45	3.40	3.41		0.154		0.579	0.652
Total	10.65	10.81	10.82		0.326		0.410	0.320
Relative width (% of total)								
Articular cartilage	30.4	32.2	31.0		0.012		0.003	0.358
Growth plate	34.5	35.2	36.6		0.013		0.737	0.025
Mineralisation zone	34.1	32.6	32.4		0.011		0.016	0.008

1 Values are from 24 animals per treatment (3 animals from 8 pens) with measurement of both legs.

2 Compared to Ctrl.

### Effects of pro and post on digestibility

The apparent digestibility showed no effects of Pro and Post on dry matter, nitrogen and ash digestibility when compared to the Ctrl (Table 3). Birds fed the Pro diet had a lower Ca and P digestibility (−8.2 % and −4.7 %, respectively) compared to the Ctrl, while Post had no effect on Ca and P digestibility. The apparent digestibility of total and insoluble NSP were not affected by Pro and Post. Dietary total and soluble NSP content were 10.2 and 1.9 % on dry matter basis, respectively. The Pro had no effect on the apparent digestibility of any of the monosaccharides. The Post resulted in a lower apparent digestibility of total galactosyl and mannosyl by 36.6 and 13.9 %, respectively, and of insoluble mannosyl by 16.3 %. Of note, the apparent digestibility of total NSP, arabinosyl, fucosyl, galactosyl, rhamnosyl and xylosyl, and of insoluble galactosyl, glucosyl, rhamnosyl and xylosyl were, on average, among all treatments negative.

**Table 3:** Apparent ileal digestibility (%)^1^ of 37-day-old male Ross 308 fed a control (Ctrl) diet supplemented with either a lactobacilli-based probiotic (Pro) or its derived postbiotic (Post) from day 1 onward.

Parameter^2^	Ctrl	Pro	Post		SEM		p-value^3^	
							Pro	Post
Dry matter	62.0	61.9	60.9		0.85		0.833	0.098
Nitrogen	68.9	69.1	69.6		1.67		0.867	0.534
Organic matter	63.3	63.3	62.1		0.86		0.917	0.099
Ash	35.8	34.3	35.1		1.20		0.143	0.519
Calcium	47.6	43.7	48.1		2.47		0.007	0.688
Phosphorus	55.6	53.0	54.3		1.65		0.036	0.238
Total NSP^4^	−3.9	−3.2	−5.5		2.71		0.659	0.340
Arabinosyl	−3.5	−3.4	−4.2		2.41		0.946	0.632
Fucosyl	−71.6	−70.4	−75.1		4.55		0.743	0.346
Galactosyl	−11.2	−11.5	−15.3		2.03		0.881	0.018
Glucosyl	−7.7	−5.2	−7.3		3.83		0.315	0.871
Mannosyl	41.6	40.7	35.8		1.63		0.379	< 0.001
Rhamnosyl	−39.5	−42.6	−33.4		5.61		0.417	0.109
Xylosyl	−8.2	−7.6	−10.3		3.58		0.731	0.296
Uronyl	1.9	0.4	−1.1		5.09		0.721	0.473
Insoluble NSP	2.2	3.4	1.3		2.50		0.500	0.586
Arabinosyl	8.6	8.5	7.3		1.77		0.924	0.292
Fucosyl	20.3	21.0	19.6		4.32		0.845	0.841
Galactosyl	1.2	−0.2	−1.6		3.18		0.589	0.284
Glucosyl	−4.3	−1.5	−3.2		3.59		0.234	0.616
Mannosyl	35.5	37.1	29.7		3.80		0.692	0.004
Rhamnosyl	−15.4	−16.4	−11.8		7.14		0.863	0.540
Xylosyl	−1.3	0.0	−3.0		3.00		0.511	0.372
Uronyl	7.3	7.4	6.9		3.12		0.984	0.869

1 As measured over the entire ileum digesta.

2 Values are the mean of 8 pen replicates per treatment with 10 broilers each.

3 Compared to Ctrl.

4 Non-starch polysaccharides.

### Effects of pro and post on diversity and structure of the caecal digesta microbiota

Sequencing of the ileal and caecal microbiota produced a total of 6,365,564 16S V3-V4 reads. After denoising, removing chimera and filtering low quality sequences, a total of 5,002,587 sequences were assigned to 722 OTUs out of which 6,608 sequences belonging to 26 OTUs were excluded based on blast coverage < 0.99 and 26,325 sequences belonging to 5 OTUs were excluded based on identity < 0.95. Minimum sequencing depth for ileal and caecal samples were 22,834 and 24,939, respectively. The final dataset contained all samples with, on average, 33,887 reads belonging to 458 OTUs in ileal, and 35,594 reads belonging to 704 OTUs in caecal samples. After filtering for OTUs present in less than 10 % of the samples of a segment, 150 and 671 OTUs remained for the ileum and caeca, respectively.

**Fig. 1:**
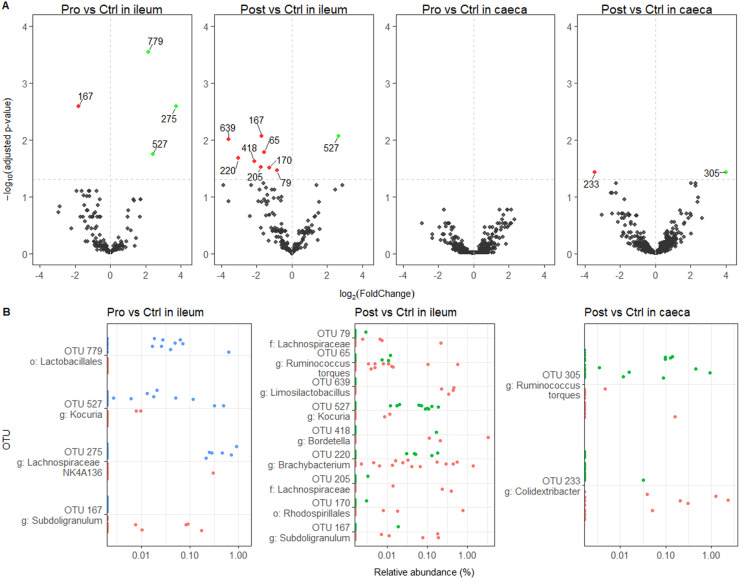
Microbial composition differences in ileal and caecal digesta of 35-day-old Ross 308 male broilers fed a control (Ctrl) diet supplemented with a lactobacilli-based probiotic (Pro) and postbiotic (Post) from day 1 onward. *N* = 24 per treatment per segment from 8 pen replicates. **(A)** Volcano plots showing the Operational Taxonomic Unit (OTUs) abundance fold change and adjusted-p for Pro *v.s*. Ctrl and Post *v.s*. Ctrl comparisons. Numbers represent the OTU ID. The horizontal dashed line represents adjusted-*p* = 0.05. Green, red and grey dots represent significantly increased OTUs, significantly depleted OTUs and not different OTUs, respectively. **(B)** Relative abundance (log scale) per samples of the differentially abundant OTUs. Each point represents an individual broiler. Red, blue and green symbols belong to Ctrl, Pro and Post, respectively. OTU identification is provided at the best identification level: o: order, f: family, g: genus.

None of the seven α-diversity (Observed, Chao1, Ace, Shannon, Simpson, inverse Simpson and Fisher) and the four β-diversity indexes (Bray, Jaccard, Unifrac and Weighed Unifrac) in both ileal and caecal digesta were affected by Pro nor Post when compared to the Ctrl (*p* > 0.1) (Supplementary Fig. 1A and B). The Pro increased the relative abundance of three OTUs and lowered the abundance of one OTU in ileal digesta, while no significant differences on OTU relative abundance in caeca digesta (Fig. 1A) were detected. The Post affected the relative abundance of nine OTUs in ileal (1 up; 8 down) and two OTUs in caecal digesta (1 up; 1 down). None of the OTUs affected by Pro nor Post was identified at the species level and their relative abundance per sample are presented in Fig. 1B. Notably, both Pro and Post increased and decreased the prevalence of the OTU 527 of genus *Kocuria* and OTU 167 of genus *Subdoligranulum*, respectively. No lactobacilli-related OTUs were found to be enriched.

### Effects of pro and post on SCFA, (iso-)caproate and semi-polar metabolome of ileal and caecal digesta

**Fig. 2:**
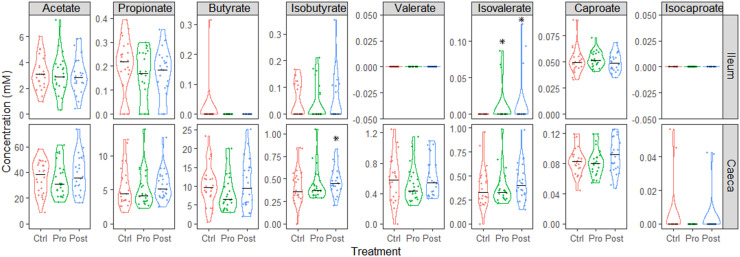
Volatile short-chain fatty acids and (iso-) caproate concentration in ileal and caecal digesta of 35-day-old Ross 308 male broilers fed a control (Ctrl) diet supplemented with a lactobacilli-based probiotic (Pro) and postbiotic (Post) from day 1 onward. Each dot represents a broiler (total 24 per treatment, 8 pen replicates). * *p* < 0.05 compared to Ctrl. The horizontal black line represents the median per treatment. Values below the limit of detection are set at 0.

The SCFA and (iso-)caproate concentrations in ileal and caecal digesta are presented in Fig. 2. The Pro and Post increased the ileal isovalerate concentration (*p* = 0.014 and 0.028, respectively) due to three values recorded for each Pro (same pen) and Post (two pens), while all others were below the level of detection. The Post decreased the caecal concentration of isobutyrate (+14.5 %; *p* = 0.032). All other SCFA were not affected (*p* > 0.1) in the two digesta locations by Pro and Post.

For the semi-polar metabolome, a total of 2,156 and 2,452 features were detected in the ileal and caecal digesta samples, respectively, of which 30 and 100 could be unambiguously annotated (Level 1), respectively. Additionally, 72 and 229 features for ileal and caecal digesta samples, respectively, were annotated by accurate mass and matched to the retention time of reference standards run on the same system (Level 2a). Also, 44 (ileal) and 158 (caecal) features could be annotated by matching them to the accurate mass and MS/MS fragmentation of reference spectra (Level 2b) with 197 and 789 features for ileal and caecal digesta samples, respectively, able to be annotated by exact mass and isotope pattern and reference count (Level 3). Finally, for ileal and caecal digesta samples, 1,813 and 1,176 features, respectively, were distinguished from the background and were not identified. The univariates statistics revealed no significant adjusted-p for Pro nor Post effects on the semi-polar metabolome in both ileum and caeca digesta (Supplementary Fig. 2A). Additionally, none of the OPLS-DA models showed significant p-values for the goodness-of-fit (pR2Y) and the predictive ability (pQ2Y) of the models for Pro and Post effects on the semi-polar metabolome in the ileal and caecal digesta (Supplementary Fig. 2B).

### Effect of pro and post on tissue transcriptome profile

**Fig. 3:**
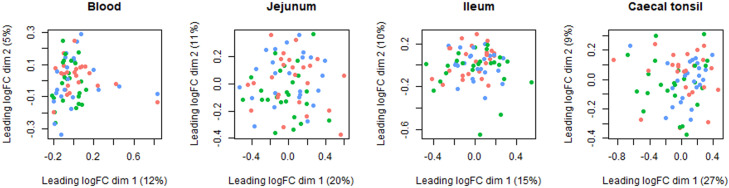
Blood, jejunum, ileum and caecal tonsil tissue transcriptome multidimensional scaling (MDS) plot of 35-day-old Ross 308 male broilers fed a control (Ctrl) diet supplemented with a lactobacilli-based probiotic (Pro) and postbiotic (Post) from day 1 onward. Each dot represents a broiler (total 24 per treatment with 8 pen replicates, but *n* = 23 for Post in ileum). Red, green and blue dots belong to Ctrl, Pro and Post, respectively.

The total number of reads per sample ranged from 23 to 139 million, with an average of 85 million reads per sample. Following the pseudo-alignment to the reference chicken transcriptome, the final aggregated counts per sample ranged from 18 to 104 million reads. In total 17,068 genes were found expressed with at least one count, out of which 15,022 remained after filtering. At the level of the global transcriptome, multidimensional scaling showed an absence of clustering between treatments in all sample types (Fig. 3).

**Fig. 4:**
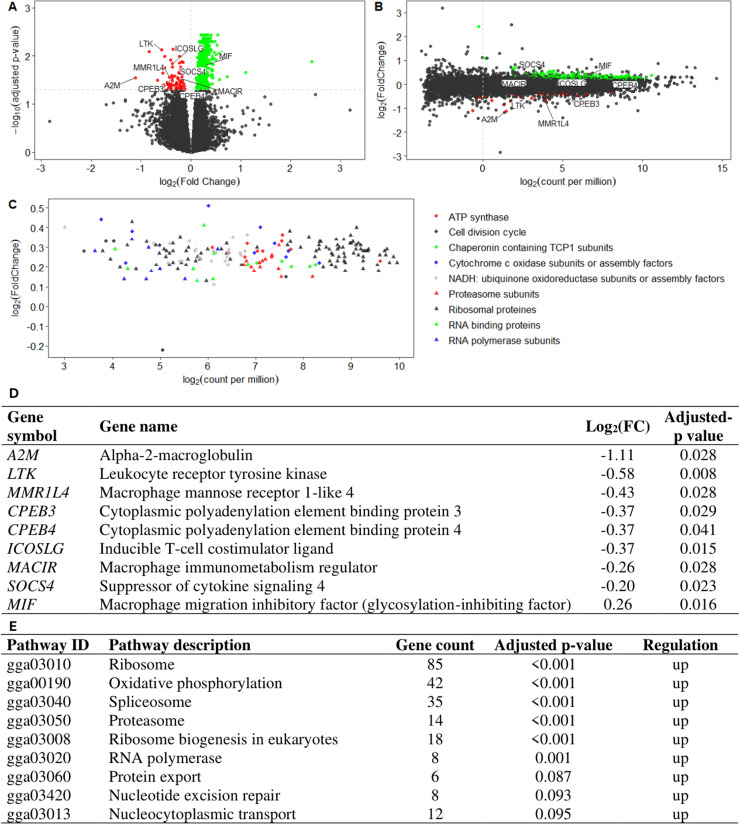
Effect of a lactobacilli-based probiotic (Pro) on the jejunum tissue transcriptome of 35-day-old Ross 308 male broilers fed a control (Ctrl) diet. *N* = 24 per treatment with 8 pen replicates. **(A)** Volcano plot showing the gene mRNA fold change and adjusted-p for Pro *v.s*. Ctrl and **(B)** associated MA plot. **(C)** Ten categories of genes with highest representation among the differentially expressed genes (DEG). **(D)** Subset of differentially expressed immune related genes. **(E)** Over representation analysis of the up- and downregulated DEG. Only upregulated DEG were associated with overrepresented pathways.

Differential analyses in each tissue showed contrasted results. While no DEG were identified for Pro and Post in the blood, ileum and caecal tonsils, in the jejunum significant differences were found but only for Pro with 654 upregulated DEG and 75 downregulated DEG (Fig. 4A and B; Supplementary Table 1). Among functional categories overrepresented in these results some gathered a high number of DEG, with all DEG except one in the represented categories being upregulated (Fig. 4C). The latter categories correspond to genes involved in energy production, DNA transcription to protein and cell multiplication. A subset of DEG known to be important in immune cells (*e.g. LTK, MMR1L4, CPEB3* and *4, SOCS4, ICOLSLG, MACIR* and *MIF*) and the inflammatory markers *A2M* are represented in Fig. 4A, B and D as examples. No pathways were over-represented in the downregulated DEG (*p* > 0.1) (data not shown). The KEGG pathway over-representation analysis revealed that the up-regulated DEG belonged to six highly significantly over-represented pathways and three pathways with *p* < 0.1 (Fig. 4E). Most pathways are related to an increase in cell activity including DNA transcription to proteins (*e.g*. ribosome, proteasome, RNA polymerase and protein export pathways), energy production (oxidative phosphorylation pathway) and resistance to oxidative stress (spliceosome pathway).

**Fig. 5:**
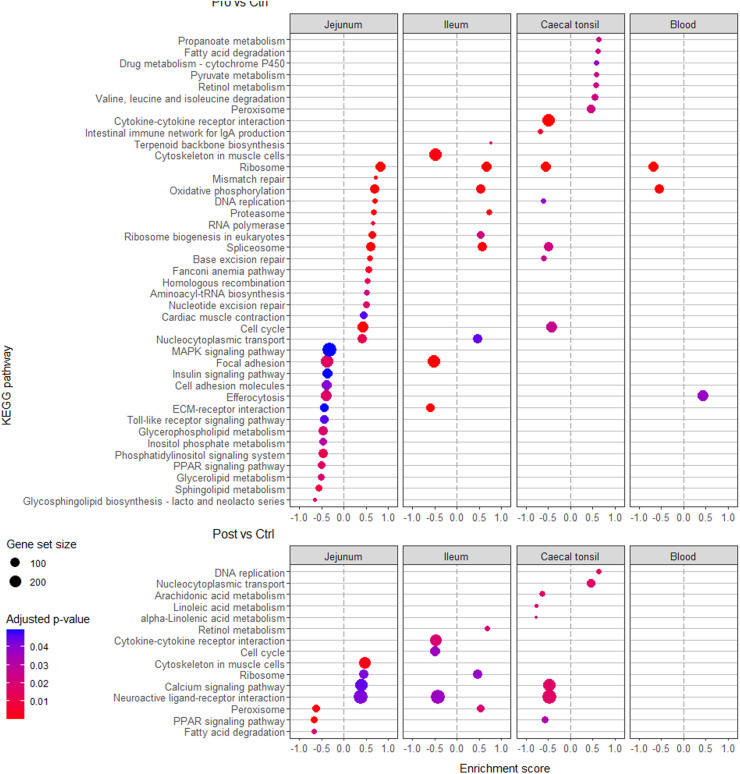
Gene set enrichment analysis of Kyoto Encyclopedia of Genes and Genomes (KEGG) pathways of the blood, jejunal, ileal and caecal tonsil tissue transcriptome of 35-day-old Ross 308 male broilers fed a control (Ctrl) diet supplemented with either a lactobacilli-based probiotic (Pro) or a postbiotic (Post) from day 1 onward. Only pathways with an adjusted-*p* < 0.05 are represented.

**Fig. 6:**
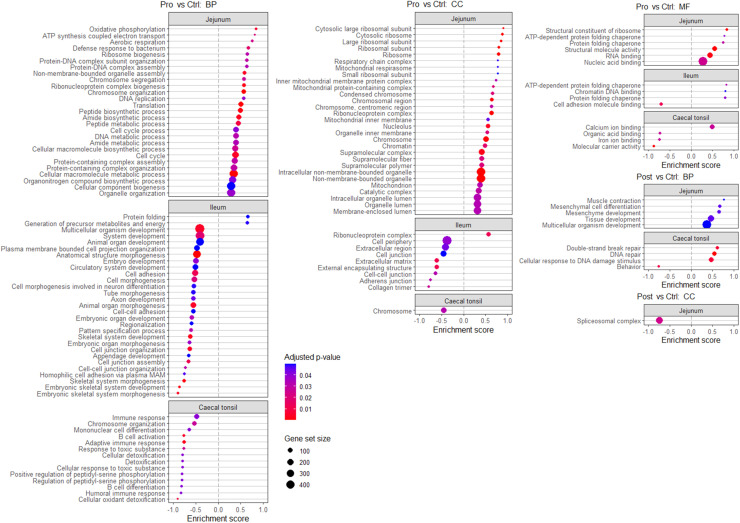
Gene set enrichment analysis of Gene Ontology (GO) for biological process (BP), cellular component (CC), and molecular functions (MF) in jejunum, ileum and caecal tonsil tissue transcriptome of 35-day-old Ross 308 male broilers fed a control (Ctrl) diet supplemented with either a lactobacilli-based probiotic (Pro) or its derived postbiotic (Post) from day 1 onward. Pathways with a false discovery rate < 0.05 are represented. A positive enrichment score indicates that the pathway was more expressed in the Pro or Post group. MAM: membrane adhesion molecules.

Complementary to the results on DEG, the GSEA for Pro and Post effects of KEGG and GO pathways evaluated if there were effects at these levels (Fig. 5, Fig. 6, respectively). In jejunal, ileal and caecal tonsils tissues, Pro affected the enrichment score of 30, 10 and 14 KEGG pathways, respectively, while for Post there were 7, 6, and 8 pathways, respectively. The Pro affected the enrichment score of 62, 42, and 19 GO pathways in jejunal, ileal and caecal tonsil tissues, respectively, while Post affected the enrichment score of 6 and 4 GO pathways in jejunal and caecal tonsil tissues, respectively. The blood transcriptome showed enriched pathways only for KEGG with Pro (3 pathways). Overall, more unique KEGG and GO pathways had a significant enrichment score for Pro *v.s*. Ctrl (41 and 118, respectively) compared to Post *v.s*. Ctrl (15 and 10, respectively).

When comparing Pro with Ctrl fed birds, pathways linked to cell activity, energy production and DNA transcription were upregulated in the jejunal tissue according to GSEA of KEGG (*e.g*. ribosome, DNA replication, RNA polymerase, cell cycle and nucleocytoplasmic transport) and GO (*e.g*. ribosome and ribosomal subunits, oxidative phosphorylation, DNA replication, cell cycle, cellular components biogenesis, RNA binding and chaperones pathways) pathways. Similar findings were observed in ileal tissue, despite a lower number of pathways that had a significant enrichment score (*e.g*. KEGG: ribosome, oxidative phosphorylation and spliceosome pathways; GO: generation of precursors metabolites and energy, ribonucleoprotein complex and chaperone pathways). In jejunal tissue, the downregulated pathways with Pro were only observed with the KEGG analysis and linked to immunity and inflammation (*e.g*. MAPK signalling, efferocytosis, toll-like receptor signalling pathways), and lipid metabolism (*e.g*. PPAR signalling, glycolipids and (glycol-)sphingolipids metabolism pathways). In both jejunal and ileal tissue, Pro downregulated the extracellular matrix (ECM)-receptor interaction and focal adhesion KEGG pathways. This effect on cell-cell interactions and junctions was more pronounced in the ileum where many GO pathways had a negative enrichment score (*e.g*. cell adhesion and morphogenesis, cell junction and cell junction organisation, adherens junction, skeletal system morphogenesis and development and cell adhesion molecule binding). In the caecal tonsil tissue, Pro was associated with negative enrichment score of immune and detoxification-related pathways in KEGG (*e.g*. cytokine-cytokine receptor interaction and intestinal immune network for IgA production pathways) and GO (*e.g*. immune response, mononuclear cell differentiation, B cell activation, humoral immune response, cellular detoxification and cellular response to toxic substance pathways). The Pro had minor effects on the blood transcriptome with only three KEGG pathways affected, namely ribosome and oxidative phosphorylation pathways with a negative enrichment score, and the efferocytosis pathway with a positive enrichment score.

When comparing the Post to Ctrl birds, Post had few significant effects on the transcriptome in jejunal, ileal and caecal tonsil tissues. The Post was associated with a positive enrichment score of GO mesenchyme development-related pathways and KEGG cytoskeleton in muscle cells and ribosome pathways. In ileal tissue, Post only affected KEGG pathways namely, the retinol metabolism, ribosome and peroxisome pathways, which were enriched and the cytokine-cytokine receptor, cell cycle and neuroactive ligand receptor interaction pathways which showed a negative enrichment score. In caecal tonsil tissue, Post was associated with a positive enrichment score of pathways related to DNA replication and repair (*e.g*. DNA replication, nucleocytoplasmic transport, DNA and double strand break repair pathways) and negative enrichment of KEGG PPAR signalling and fatty acids metabolism (arachidonic, linoleic, and α-linoleic acid) pathways.

## Discussion

It has previously been reported that at d35, Pro but not Post increased broiler BW while Pro and Post had no effect and increased FCR, respectively (Jansseune et al., 2025). Some effects on the blood plasma concentration of nutrition-related biochemical parameters and immune cells concentrations were also observed. The current study aimed to provide a more in-depth investigation into *in vivo* effects of Pro and Post, and reports results on apparent ileal digestibility and leg health, as well as on ileal and caecal digesta microbiota, volatile fatty acids, and semi-polar metabolome composition to more clearly identify effects of Pro and Post on broiler physiology. Effects of Pro and Post on the metabolism were investigated by RNA-seq of the jejunum, ileum, and caecal tonsil tissues as well as in the blood.

The main effect of Pro appears to occur in the proximal part of the gastro-intestinal tract as most effects on the transcriptome were localised in the jejunal compared to the ileal and caecal tonsil tissues. Also, the minor effects of Pro on the microbiota were localised in the ileum but not in the caeca. Similarly, Post affected the relative abundance of more OTUs in the ileal than in the caecal digesta. These very limited effects on the microbiota may not have resulted in important changes on their activity as the semi-polar metabolome analyses showed no significant differences between treatments. Unfortunately, the microbiota composition could not be measured in jejunal digesta, due to the low quantity of DNA extracted, thereby indicating that per unit of digesta, the number of bacteria present in jejunal digesta were low compared to the ileal and caecal digesta. Possibly due to this lower microbial density, a greater impact of a probiotic can be expected when it comes to eliciting biological effects.

The positive impact on broiler growth of Pro in the absence of a significant modification of microbiota composition and metabolomic profile of the gut content, oppose many findings where the beneficial effects of probiotics on broiler growth were argued to result from the concomitant modification of the microbiota composition and/or metabolome (Danladi et al., 2022a, b; Chai et al., 2023; Yin et al., 2023; Yang et al., 2023, 2024). Our study indicates that probiotics can elicit beneficial growth promoting effect in broilers in the absence of a modification of the microbiota composition and activity. In the current experiment it likely occurred through a reduction of intestinal inflammation as suggested by the enrichment analysis of KEGG and GO pathways. The KEGG and GO pathways analyses provides valuable information on the potential functional role of DEG. However, there are also inherent limitations to this approach such as incomplete mapping, the functional interaction between genes being ignored, and dependence on sequenced and filtered genes (Tamayo et al., 2016). Other downstream mechanisms may be affected such as mRNA transcription and transduction or enzyme activity. Nevertheless, pathways enrichment analyses allows broad screening of biological processes and identification of potential modes of action (García-Campos et al., 2015).

The GSEA of KEGG and GO pathways indicated that Pro lowered inflammatory and immune-related pathways in jejunal and caecal tonsil tissue. This effect may have been greater in the jejunum as indicated by the higher number of differentially enriched pathways compared to the ileum and by the significant differential expression of immune related genes. A reduced expression of intestinal inflammatory markers was also reported in broilers supplemented with the probiotic *L. rhamnosus* GG (Zhang et al., 2024), *L.* paracasei LK01 (Liu et al., 2024) and *L. johnsonii* BS15 (Wang et al., 2017a), or a three strain probiotic cocktail (Gao et al., 2024). However, the modes of action of probiotics are strain and challenge specific which limits the possibility for detailed comparisons between studies (McFarland et al., 2018).

Notably, the gene with the most reduced expression in Pro compared to Ctrl was *A2M* indicating a reduced inflammation. In humans and mice, *A2M* has been described as a versatile proteinase having many roles in inflammation and produced as an acute phase protein (Vandooren and Itoh, 2021; Lagrange et al., 2022). Other immune related gene with altered expression by Pro included *SOCS4, LTK, ICOSLG, CPEB3* and *4, LTK*, and *MACIR* which mRNA expressions were downregulated, and *MIF* which expression was upregulated. *SOCS4* is broadly expressed by cells of the immune system and is a modulator of T cells (Luckey et al., 2011; Kedzierski et al., 2014). To the best of our knowledge, the *ICOSL* gene has not been studied in chickens, but in human where it was associated with T cell polarisation and B cell immunity and influences intestinal inflammation (Li and Xiong, 2020). The *CPEB4* gene was reported to be chronically overexpressed in inflammatory cells in patients with chronic intestinal inflammation (Sibilio et al., 2022). The CPEB3 was reported to support the recruitment of regulatory T cells and lowering the recruitment of immune cells with anti-cancer activity in renal carcinoma (Hong et al., 2024), and of anti-inflammatory macrophages in colorectal carcinoma (Zhong et al., 2020). Although the precise biological role of the leucocyte receptor tyrosine kinase (**LTK**) protein remains to be clarified, it is expressed in multiple tissues (Cooper et al., 2022), and has been reported to induce the recruitment and activation of leukocytes (Szilveszter et al., 2019). Interestingly, compared to the other genes, *MIF* expression was increased with Pro in jejunal tissue, as in chickens, MIF protein effects include inhibition of macrophage migration, enhancement of inflammatory response of monocytes, and proliferation of activated T lymphocytes (Kim et al., 2010). Another regulator of macrophage function is the MACIR protein, through enhancement of the resolution of inflammation and repair functions (Serwin et al., 2023). Lower *MACIR* expression, in the present study, could indicate a lower need for the resolution of inflammation in the jejunal tissue of birds receiving Pro.

The lower expression of inflammatory and immune pathways as well as specific expression of markers of immune cells by Pro could be linked to a lower inflammation in the jejunum of Pro-fed birds compared to birds receiving the Ctrl diet. This may be associated with a lower infiltration of the jejunum by immune cells. Indeed, gut infiltration by immune cells is increasing with the need to fight pathogens and associated inflammation (Cardoso Dal Pont et al., 2023). This is also indicated by the lower expression of *MMR1L4*, which is expressed by monocytes and macrophages, encodes a mannose receptor and serves as a marker to identify those cells from other immune cells (Wu et al., 2023; Maxwell et al., 2024). As RNAseq analyses are performed on an equal RNA quantity per sample basis and a normalisation of gene mRNA counts, the greater expression of pathways linked to cell activity, energy production and DNA transcription may result from a greater representation of epithelial cells in the samples. In the ileum, Pro treatment was associated with a negative enrichment of pathways associated with the development of this tissue, including extracellular matrix synthesis and cell development, which indicates a lower rate of development of the ileum or the need for less tissue synthesis resulting from a lower cell turnover. A greater development or activity of the jejunum and ileum epithelial cells could result in an improved nutrient digestibility which was not observed. This would support the thesis that the effect on cell activity and development in jejunal and ileal tissues were likely due to a greater presence of epithelial cells and reduced cell turnover, respectively.

A lower activation of the immune system may have also occurred in the caeca as shown by the transcriptomic profile of the tonsils. The caecal tonsils are a lymphatic tissue which are particularly relevant when studying immune-related processes in caeca (Haghighi et al., 2008; Setta et al., 2012; Cazals et al., 2022) and because of their role in immunity of broilers, the tonsils may have a high immune sensitivity. As in our study Pro had no significant effect on the caecal microflora and metabolome content, it suggests that the effect of Pro on the tonsils could have been induced by a reduction of the inflammatory signals produced from the jejunum, while the absence of effects on the ileum and blood transcriptome profile related to immunity may result from a lower sensitivity of these tissues. Accordingly, Cardoso Dal Pont et al. (2023) reported that the jejunum was reported to be more responsive than the ileum in an inflammation model using high dietary NSP levels in broilers.

Broilers are exposed to multiple factors that can promote inflammation in the intestine, such as pathogens, certain feed ingredients, high energy diets (Kogut et al., 2018), and high stocking density (Hafez et al., 2022) besides temperature and stress (Tellez et al., 2023). Particularly, soybeans have been reported to contain components with pro-inflammatory effects in broilers (Belal, 2020), pigs (Wang et al., 2023c), mice (Sun et al., 2013) and fish (Li et al., 2017a), while diets rich in NSP were reported to promote intestinal inflammation in broilers (Chen et al., 2015; van Krimpen et al., 2017; Cardoso Dal Pont et al., 2021). A relatively higher level of NSP was used in the diet of the current experiment which was also found to impair broiler growth performance parameters (Jansseune et al., 2024b, c). The Pro did not affect NSP digestibility, indicating that its beneficial effects on growth and inflammation do probably not originate from a direct effect on NSP. Lactobacilli probiotics can also have a direct influence on host epithelial and immune cells by lowering inflammation and can produce cellular components with immune-modulatory properties [*e.g*. peptidoglycan and (exo-)polysaccharides) (Morshedi et al., 2019; Angelin and Kavitha, 2020; Dempsey and Corr, 2022)]. The effects of probiotic bacteria are strain and species specific, thereby, limiting the possibility for extrapolation of effects (Marteau, 2011). The bacteria in Pro were reported for their ability to bind to an *in vitro* model of intestinal epithelium (Bernardeau et al., 2001). This may activate an anti-inflammatory response in host cells or reduce the adherence sites for other bacteria. Thus, the lower inflammation in the jejunum observed here can results from either a modulation of the microbiome, its activity and/or from a direct effect of Pro bacteria or their components on host cells. Reduction in intestinal inflammation was proposed to be the potential primary mechanism of antibiotic growth promoters, and the strategy for their replacement (Niewold, 2007, 2014), with numerous studies reporting anti-inflammatory benefits of alternatives as reviewed by Broom and Kogut (2018). The lower gut inflammation was not associated with improved nutrient digestibility but may have allowed for a greater feed intake of the birds, thereby, promoting growth. To the best of our knowledge, correlation between voluntary feed intake and intestinal inflammation has not been investigated in broilers, but challenges which induce inflammation (*e.g*. immune and heat stress) were reported to reduce feed intake (Remus et al., 2014; Liu et al., 2020). Challenges inducing inflammation likely also involve additional anorexigenic mechanisms. The absence of an effect of Pro on the apparent digestibility of nitrogen indicates that the broilers did not increase their net amino acid absorption. Together with the absence of effects on blood plasma uric acid concentration for Pro at d35 (Jansseune et al., 2025) which results from amino acids catabolism, it appears that protein absorption and deposition rate were not affected by Pro. This would lend support to Pro-induced growth being mostly related to a greater feed intake.

A reduced inflammation associated with the Pro treatment may have also contributed to the observed Pro-associated increase in tibia articular cartilage width. Cartilage homeostasis is maintained and controlled by chondrocytes, while local and possibly systemic inflammation can disturb cartilage homeostasis leading to osteoarthritis, including cartilage breakdown (Rahman et al., 2023). Some probiotics can slowdown induced osteoarthritis (cartilage breakdown) in association with reduced local inflammation (Rahman et al., 2023). Also, some exogenous molecules can support cartilage growth and extracellular matrix synthesis (Li et al., 2020), with a possible involvement of the microbiota (Hao et al., 2021). The effects of Pro may also have been partly mediated directly by some metabolites in the Pro biomass as Post also increased the articular cartilage width. Thus, a Pro-associated increase of articular cartilage width may result from a combination of reduced systemic inflammation and effect of metabolites. The Pro-associated increase of articular cartilage width reported here was previously observed for Pro with its commercial carrier (Jansseune et al., 2024c), which would indicate that this effect is due to Pro and not the carrier. This repeatable observation of Pro increasing articular cartilage width in broilers could be of interest in osteoarthritis research in humans and other animal species suffering this condition such as cats and dogs. Surprisingly, the articular cartilage was also found to be affected by leg location, whereby, the right leg had a wider cartilage, which may result from the reported right footedness observed in broiler chickens (Rogers and Workman, 1993; Tommasi and Vallortigara, 1999). For bone growth and health, Ca and P are particularly important (Proszkowiec-Weglarz and Angel, 2013). As no leg associated problems were observed in our study, the supply of nutrients important for bone health including Ca and P were unlikely to be limiting, despite the ileal Ca and P digestibility being reduced by Pro (Table 2).

The observation that Post induced an increase in TD score contradicts our previous findings (Jansseune et al., 2024c) where Post with its commercial carrier lowered the TD score in broilers. Here the presence of sepiolite (Mg₄Si₆O₁₅₂·6H₂O) in the carrier would likely explain the difference. Tibia dyschondroplasia corresponds to an abnormal mineralisation leading to an abnormal enlargement and deformation of the growth plate. The mechanisms underlying TD remain poorly understood but have been associated with a deficiency in nutrients essential for bone growth, oxidative stress and inflammation in the growth plate (Dong et al., 2022). Why the Post treatment in the present study had a deleterious effect on TD score remains difficult to explain and why the opposite was observed when the carrier was present. Possibly the Mg present in the sepiolite, which has been shown to ameliorate chloride-induced dyschondroplasia in broilers (Luo et al., 1992) may have prevented an increase in TD score when the Post with its carrier was supplemented. Why Post, and not Pro, increased TD score in the present study remains to be explained.

In the current experiment, Pro affected the host’s metabolism more compared to Post, as indicated by the number of KEGG and GO pathways with significant enrichment score, and immune cell counts (Jansseune et al., 2025), despite Post being supplemented at a concentration 10x greater than Pro. The major difference between Pro and Post is the presence of viable bacteria in Pro and the high temperature short time (**HTST**) treatment of Post. As such, it would be logical to conclude that the viable bacteria are responsible for the effects of Pro but not, or to a far lesser extent, the cell components or the metabolites. Alternatively, the HTST treatment of Pro to derive Post altered the beneficial components present in Pro. Lactobacilli peptidoglycans are reported to elicit biological effects (Clarke et al., 2010; Arentsen et al., 2017; Huang et al., 2020) but can be heat sensitive (Wang et al., 2023a) with intact and altered peptidoglycan having different effects (Huang et al., 2020). Lactobacilli (exo-)polysaccharides were also extensively reviewed for their biological effects (Segers and Lebeer, 2014; Patten and Laws, 2015; Castro-Bravo et al., 2018; Angelin and Kavitha, 2020), and their structure can be altered by technological treatment (Nachtigall et al., 2021). Inactivation of live microbes in Pro without a heat treatment may be done by UV radiation, sonication or high pressure processing (Meena et al., 2025). A less likely option is an overdosing of Post, as in that case, greater biological effects on the host would be expected but not resulting in beneficial effects.

Overall, Post had limited effects on the digesta microbiota and volatile fatty acids, as well as on the tissue transcriptome, and had no effects on the semi-polar metabolome in digesta. Furthermore, no differentially abundant OTUs in digesta were identified at a species level and the tissue transcriptomic data did not show a clear pattern of the mechanism(s) associated with Post effects. Accordingly, some pathways were differentially affected by Post between the jejunal, ileal, and caecal tonsil tissues, namely the calcium-signalling, neuroactive ligand-receptor interaction and peroxisome pathways. Nevertheless, Post may have influenced lipid metabolism in the jejunal and caecal tonsil tissues. In the jejunal tissue, Post lowered the expression of the peroxisome, PPAR signalling and fatty acids degradation pathways and in caecal tonsil tissue, the expression of the PPAR signalling and arachidonic, linoleic and α-linoleic acids metabolism pathways. Under the experimental conditions and design of the study, no clear biological effects of Post were observed, although some parameters and pathways were modulated.

The digestibility of protein was not affected by Post while blood plasma uric acid concentration was increased at d35. This is in line with the higher FCR at d35 which suggests that a greater catabolism of amino acids occurred or there was an imbalance in nutrient uptake by birds receiving Post. Increase in FCR with Post can result from the trend observed in a lower digestibility of dry and organic matter (Table 2). Energy was likely not limiting for growth which is supported by the blood cholesterol data. Cholesterol is one of the two intermediates synthetised by the liver for the storage of glucose as fat in adipose tissue (Wang et al., 2017b) and was found to be increased at d35 (Jansseune et al., 2025). An increase in blood cholesterol can also be due to a reduced catabolism of cholesterol. The Post lowered the apparent ileal digestibility of some NSP constituent sugars. The NSP constituents may be derived from endogenous sources or synthesised by the microbiota. For example, mannosyl residues are also found in mucins, and its molar ratio to the other mucin monosaccharides can be affected by feed additive supplements in broilers (Tsirtsikos et al., 2012a, b). Polysaccharides are also ubiquitous components of bacteria as constituents of cell wall *e.g*. lipopolysaccharides, peptidoglycan, capsular polysaccharides and exopolysacharides (Knirel and Valvano, 2013). As such, the NSP digestibility results should be interpreted with care as many were negative, indicating a net addition to the digesta or additional consumption through ingestion of litter material by the broilers.

## Conclusion

Different physiological effects were elicited by Pro supplementation to a broiler diet which were not observed for its derived Post. Tissue transcriptomic analyses indicated that Pro lowered inflammation in the gut with a possible reduction of systemic inflammation, thereby promoting gut health and growth performance. The effects of Pro may have originated mainly from effects on host cells in the proximal intestine, while an alteration of the microbiota and metabolome appears less likely or limited. The effects of Pro may result from the presence of viable bacteria and/or from the non-denaturation of beneficial compounds, which were absent in Post. The proximal small intestine may be more sensitive to probiotic effects, possibly due of a lower microbial load.

## Data availability

The sequencing data analysed during the current study are available in the NCBI Sequence Read Archive (SRA) database under the Bioproject accession numbers PRJNA1153316 and SUB15052620. The other datasets used and analysed in the current study are available from the corresponding author on reasonable request.

## Funding

This study was funded by Idena (Sautron, France), and the “Association Nationale Recherche Technologie” (ANRT) through the Convention Industrielle de Formation par la RecherchE (CIFRE) grant no. 2021-0384. The funding bodies played no role in the design, analysis and reporting of the study.

## CRediT authorship contribution statement

**Samuel C.G. Jansseune:** Writing – review & editing, Writing – original draft, Visualization, Supervision, Resources, Project administration, Methodology, Investigation, Funding acquisition, Formal analysis, Data curation, Conceptualization. **Wouter H. Hendriks:** Writing – review & editing, Validation, Supervision, Project administration, Conceptualization. **Fany Blanc:** Writing – review & editing, Supervision, Project administration, Methodology, Investigation, Conceptualization. **Jordi Estellé:** Writing – review & editing, Supervision, Resources, Formal analysis, Data curation. **Nicolas Bruneau:** Project administration, Investigation. **Jean-Luc Coville:** Project administration, Formal analysis. **Marie-Hélène Pinard-van der Laan:** Writing – review & editing, Supervision, Investigation, Conceptualization.

## Disclosures

Although one of the authors (SCGJ) was a PhD candidate employed by Idena, the authors attest that they were completely free to independently design the study and collect, analyse and interpretate the data as well as write the manuscript.

## Appendix Supplementary materials

Image, image 1

Image, image 2

Image, application 3

Image, application 4

## Footnotes

Supplementary material associated with this article can be found, in the online version, at doi:10.1016/j.psj.2025.106159.
