# Supplementary figures and images for: A lactobacilli-based probiotic but not its postbiotic reduces intestinal inflammatory pathways expression in broilers fed a non-starch polysaccharide rich challenge diet
Source: Poult Sci. 2025 Nov 26;105(1):106159. doi: 10.1016/j.psj.2025.106159 (PMC12723048; doi:10.1016/j.psj.2025.106159)

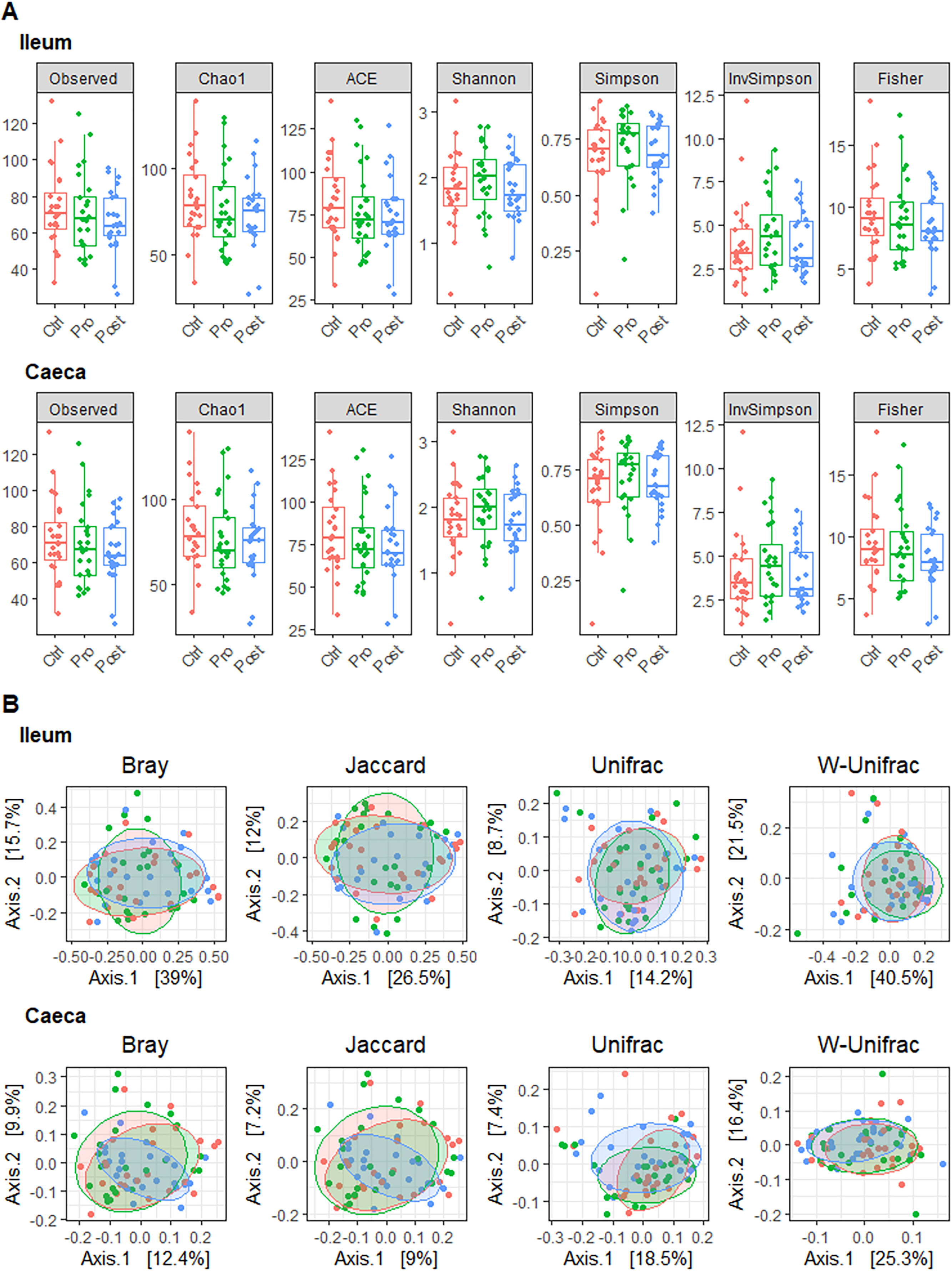

Supplement: Supplementary file 1 [file mmc1.jpg]

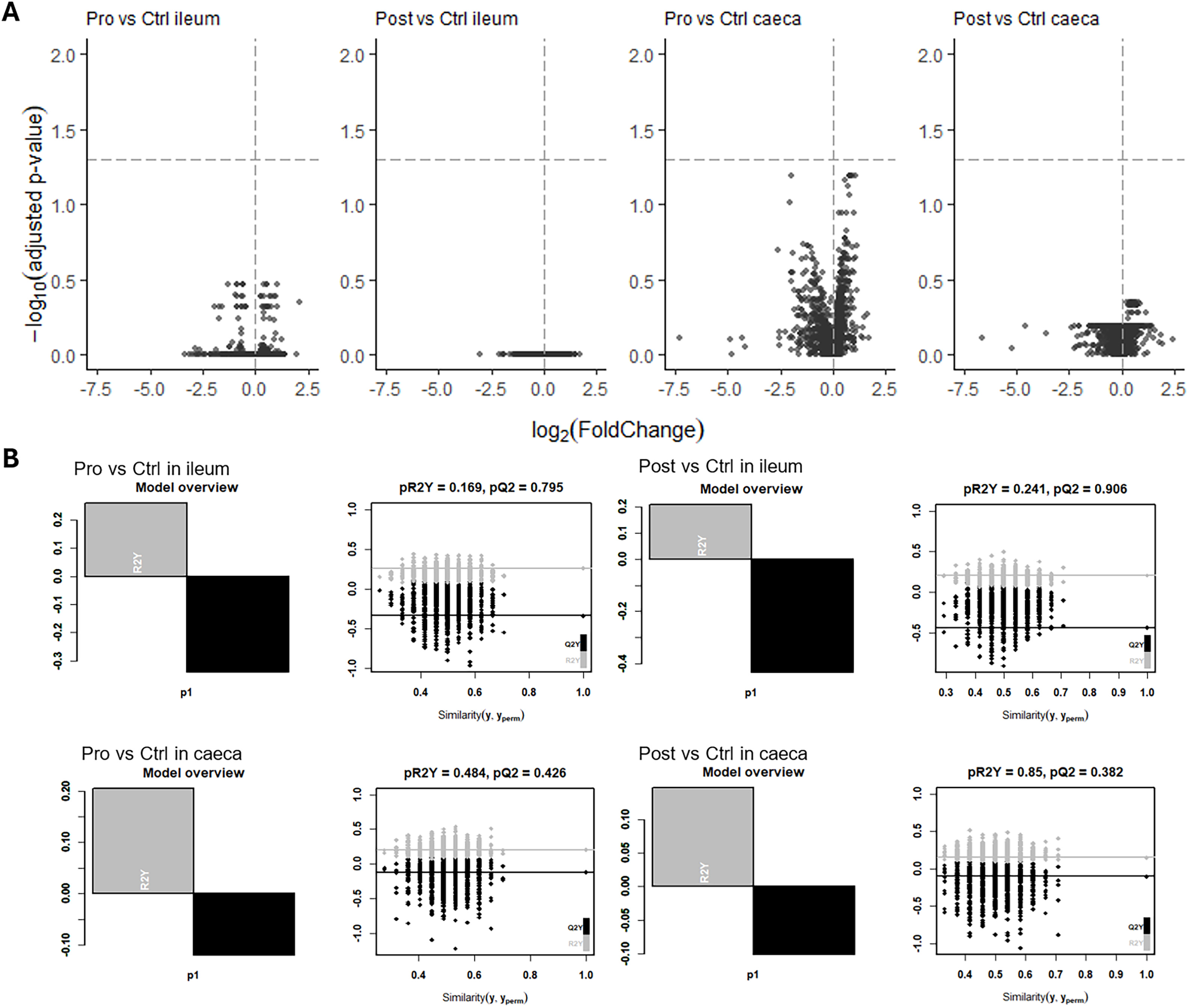

Supplement: Supplementary file 2 [file mmc2.jpg]
