# Supplementary material for: A lactobacilli-based probiotic but not its postbiotic reduces intestinal inflammatory pathways expression in broilers fed a non-starch polysaccharide rich challenge diet
Source: Poult Sci. 2025 Nov 26;105(1):106159. doi: 10.1016/j.psj.2025.106159 (PMC12723048; doi:10.1016/j.psj.2025.106159)
Supplement: Supplementary file 3 [file mmc3.docx]

**SUPPLEMENTARY INFORMATION**

**Supplementary Table 1.** Differentially expressed genes symbol, name Entrez identification number (EID), false discovery rate (FDR), log_2_ fold change (FC) and count per million (CPM) in the jejunal tissue of 35 days-old male Ross 308 broilers fed a control diet supplemented or not with a *Lactobacilli*-based probiotic.

**Supplementary Figure 1.** Microbial diversity in ileal and caecal digesta of 35-day-old Ross 308 male broilers fed a control (Ctrl) diet supplemented with either a lactobacilli-based probiotic (Pro) or its derived postbiotic (Post) from day 1 onward. **(A)** Alpha and **(B)** Beta diversity indexes. Each point represents an individual broiler. Red, green and blue symbols belong to Ctrl, Pro and Post, respectively. ACE: Abundance-based coverage estimator, InvSimpson: Inverse Simpson, W-Unifrac: Weighted unifrac.

**Supplementary Figure 2.** Effect of a lactobacilli-based probiotic (Pro) and its derived postbiotic (Post) supplemented to a control (Ctrl) diet on the semi-polar metabolome in ileal and caecal digesta of 35-day-old Ross 308 male broilers. The Pro and Post were supplemented from day 1 onward. N = 24 from broilers equally originating from 8 pens. **(A)** Volcano plot showing features fold change of Pro and Post compared to Ctrl and associated adjusted-p. Each point represents an individual feature. Grey dots represent not different features (adjusted-p > 0.05). **(B)** Orthogonal Partial Least Square-Discriminant Analysis (OPLS-DA). The relative abundance was pre-processed by missing value imputation, log^10^ transformation and Pareto scaling. Left panel (Model overview): cumulative model inertia captured by orthogonal components. R2Y (grey) and Q2Y (black) are the goodness-of-fit and the predictive performance of the model, respectively. Right panel: permutation test (1000 random permutation), the x-axis is the similarity to the original model and the y-axis represents the values of R2 and Q2. pR2Y: p value of the goodness of fit of the model and pQ2Y: p value of the predictive performance of the model.
