# Supplementary material for: A lactobacilli-based probiotic but not its postbiotic reduces intestinal inflammatory pathways expression in broilers fed a non-starch polysaccharide rich challenge diet
Source: Poult Sci. 2025 Nov 26;105(1):106159. doi: 10.1016/j.psj.2025.106159 (PMC12723048; doi:10.1016/j.psj.2025.106159)
Supplement: Supplementary file 4 [file mmc4.docx]

| **Supplementary Table 1.** Differentially expressed genes symbol, name Entrez identification number (EID), false discovery rate (FDR), log_2_ fold change (FC) and count per million (CPM) in the jejunal tissue of 35 days-old male Ross 308 broilers fed a control diet supplemented or not with a *Lactobacilli*-based probiotic. | | | | | |
| --- | --- | --- | --- | --- | --- |
| **Gene symbol** | **Name** | **EID** | **FDR** | **FC** | **CPM** |
| *A2M* | Alpha-2-macroglobulin | 418251 | 0.028 | -1.11 | 1.45 |
| *CHODL* | Chondrolectin | 418474 | 0.018 | -1.08 | -0.63 |
| *C4orf45* | C4orf45 homolog | 769822 | 0.008 | -0.84 | 1.31 |
| *ZBTB32* | Zinc finger and BTB domain containing 32 | 419759 | 0.047 | -0.7 | 1.71 |
| *WEE2* | WEE2 oocyte meiosis inhibiting kinase | 427918 | 0.032 | -0.67 | 0.57 |
| *TRPM6* | Transient receptor potential cation channel subfamily M member 6 | 100859603 | 0.046 | -0.65 | 3.92 |
| *LTK* | Leukocyte receptor tyrosine kinase | 423216 | 0.008 | -0.58 | 3.05 |
| *SLC22A15* | Solute carrier family 22 member 15 | 418336 | 0.023 | -0.56 | 2.42 |
| *CLCN4* | Chloride voltage-gated channel 4 | 428005 | 0.04 | -0.55 | -0.2 |
| *TTBK2* | Tau tubulin kinase 2 | 423241 | 0.017 | -0.53 | 0.15 |
| *PDE8B* | Phosphodiesterase 8B | 427645 | 0.01 | -0.52 | -0.43 |
| *ELOVL2* | ELOVL fatty acid elongase 2 | 420858 | 0.036 | -0.48 | 0.2 |
| *CDYL2* | Chromodomain Y-like 2 | 425886 | 0.04 | -0.45 | 3.93 |
| *ETNK1* | Ethanolamine kinase 1 | 418196 | 0.048 | -0.45 | 5.96 |
| *SLC4A10* | Solute carrier family 4 member 10 | 424188 | 0.039 | -0.44 | 2.19 |
| *MMR1L4* | Macrophage mannose receptor 1-like 4 | 771888 | 0.028 | -0.43 | 3.75 |
| *NDRG1* | N-myc downstream regulated 1 | 420321 | 0.042 | -0.43 | 5.63 |
| *ACSS1B* | Acyl-CoA synthetase short-chain family member 1B | 423347 | 0.038 | -0.41 | 1.84 |
| *FAM126B* | Family with sequence similarity 126 member B | 429010 | 0.012 | -0.41 | 5.33 |
| *EGR1* | Early growth response 1 | 373931 | 0.042 | -0.4 | 6.51 |
| *AMPD3* | Adenosine monophosphate deaminase 3 | 423041 | 0.026 | -0.39 | 5.12 |
| *PIGO* | Phosphatidylinositol glycan anchor biosynthesis class O | 101748924 | 0.048 | -0.39 | 4.6 |
| *CPEB3* | Cytoplasmic polyadenylation element binding protein 3 | 423816 | 0.029 | -0.37 | 5.06 |
| *CPEB4* | Cytoplasmic polyadenylation element binding protein 4 | 416363 | 0.041 | -0.37 | 5.23 |
| *DUSP7* | Dual specificity phosphatase 7 | 415891 | 0.033 | -0.37 | 6.55 |
| *ICOSLG* | Inducible T-cell costimulator ligand | 395968 | 0.015 | -0.37 | 4.94 |
| *ITGB8* | Integrin subunit beta 8 | 395470 | 0.017 | -0.37 | 4.17 |
| *LRRN3* | Leucine rich repeat neuronal 3 | 770169 | 0.007 | -0.36 | 3.49 |
| *SH3BP2* | SH3 domain binding protein 2 | 422883 | 0.04 | -0.33 | 4.56 |
| *RRAGD* | Ras-related GTP binding D | 107053109 | 0.026 | -0.32 | 5.66 |
| *CCNG2* | Cyclin G2 | 422512 | 0.05 | -0.31 | 6.32 |
| *MAST4* | Microtubule associated serine/threonine kinase family member 4 | 427169 | 0.048 | -0.3 | 3.6 |
| *HPS5* | HPS5 biogenesis of lysosomal organelles complex 2 subunit 2 | 107048993 | 0.05 | -0.29 | 8.12 |
| *SLC27A6* | Solute carrier family 27 (fatty acid transporter), member 6 | 769933 | 0.045 | -0.29 | 1.47 |
| *PREPL* | Prolyl endopeptidase-like | 421405 | 0.036 | -0.28 | 2.89 |
| *TBCEL* | Tubulin folding cofactor E like | 429536 | 0.037 | -0.28 | 4.46 |
| *EXOC6B* | Exocyst complex component 6B | 422955 | 0.039 | -0.27 | 3.39 |
| *FAM91A1* | Family with sequence similarity 91 member A1 | 770621 | 0.029 | -0.26 | 5.37 |
| *MACIR* | Macrophage immunometabolism regulator | 768501 | 0.028 | -0.26 | 3.22 |
| *MCF2L* | MCF.2 cell line derived transforming sequence like | 418748 | 0.049 | -0.25 | 4.32 |
| *OSBPL3* | Oxysterol binding protein like 3 | 428432 | 0.023 | -0.24 | 3.71 |
| *ZNF654* | Zinc finger protein 654 | 418428 | 0.022 | -0.24 | 5.1 |
| *KDM6A* | Lysine demethylase 6A | 418556 | 0.01 | -0.23 | 7.16 |
| *LOC107050163* | Probable very-long-chain enoyl-CoA reductase art-1 | 107050163 | 0.045 | -0.23 | 5.04 |
| *PIAS1* | Protein inhibitor of activated STAT 1 | 427514 | 0.039 | -0.23 | 7.75 |
| *TAPT1* | Transmembrane anterior posterior transformation 1 | 422824 | 0.033 | -0.23 | 6.25 |
| *CDC14B* | Cell division cycle 14B | 427473 | 0.022 | -0.22 | 5.05 |
| *PLEKHF2* | Pleckstrin homology and FYVE domain containing 2 | 420234 | 0.014 | -0.22 | 5.9 |
| *GLTSCR1L* | GLTSCR1 like | 428568 | 0.047 | -0.21 | 5.34 |
| *PIP4P1* | Phosphatidylinositol-4,5-bisphosphate 4-phosphatase 1 | 121108716 | 0.035 | -0.21 | 4.42 |
| *SSFA2* | Sperm specific antigen 2 | 424118 | 0.043 | -0.21 | 6.77 |
| *ZBTB21* | Zinc finger and BTB domain containing 21 | 427986 | 0.019 | -0.21 | 6.02 |
| *ANKS1A* | Ankyrin repeat and sterile alpha motif domain containing 1A | 419898 | 0.017 | -0.2 | 5.21 |
| *RFKL* | Riboflavin kinase | 431449 | 0.035 | -0.2 | 5.81 |
| *SOCS4* | Suppressor of cytokine signaling 4 | 423557 | 0.023 | -0.2 | 4.2 |
| *WASHC4* | WASH complex subunit 4 | 418077 | 0.042 | -0.19 | 4.46 |
| *DENND1A* | DENN domain containing 1A | 417102 | 0.013 | -0.18 | 4.4 |
| *ZNF518A* | Zinc finger protein 518A | 428964 | 0.027 | -0.18 | 4.95 |
| *CHFR* | Checkpoint with forkhead and ring finger domains | 416792 | 0.032 | -0.17 | 5.36 |
| *PRR14L* | Proline rich 14-like | 416957 | 0.036 | -0.17 | 6.51 |
| *TBC1D1* | TBC1 domain family member 1 | 426162 | 0.046 | -0.17 | 5.25 |
| *DGKE* | Diacylglycerol kinase epsilon | 770911 | 0.014 | -0.16 | 4.87 |
| *WDR7* | WD repeat domain 7 | 426855 | 0.038 | -0.14 | 5.31 |
| *OSBPL2* | Oxysterol binding protein like 2 | 419227 | 0.042 | -0.13 | 6.36 |
| *INO80* | INO80 complex subunit | 423210 | 0.049 | -0.12 | 6.36 |
| *AARS2* | Alanyl-tRNA synthetase 2, mitochondrial | 421436 | 0.05 | 0.11 | 5.52 |
| *DROSHA* | Drosha ribonuclease III | 420911 | 0.045 | 0.11 | 4.1 |
| *LARS* | Leucyl-tRNA synthetase | 416347 | 0.028 | 0.11 | 6.02 |
| *NDUFAF1* | NADH:ubiquinone oxidoreductase complex assembly factor 1 | 423214 | 0.029 | 0.11 | 6.12 |
| *PRPF3* | Pre-mRNA processing factor 3 | 426667 | 0.032 | 0.12 | 6.73 |
| *DFFA* | DNA fragmentation factor subunit alpha | 419451 | 0.031 | 0.13 | 4.56 |
| *HARS1* | Histidyl-tRNA synthetase 1 | 416132 | 0.05 | 0.13 | 6.48 |
| *LUC7L2* | LUC7 like 2, pre-mRNA splicing factor | 417971 | 0.047 | 0.13 | 6.79 |
| *MRPS34* | Mitochondrial ribosomal protein S34 | 426987 | 0.011 | 0.13 | 5.96 |
| *MAPKAP1* | Mitogen-activated protein kinase associated protein 1 | 395627 | 0.03 | 0.13 | 4.77 |
| *NARFL* | Nuclear prelamin A recognition factor like | 416535 | 0.045 | 0.13 | 6.34 |
| *RBM15B* | RNA binding motif protein 15B | 770664 | 0.031 | 0.13 | 5.77 |
| *SEC11A* | SEC11 homolog A, signal peptidase complex subunit | 415334 | 0.03 | 0.13 | 5.49 |
| *THOC5* | THO complex 5 | 417019 | 0.023 | 0.13 | 6.7 |
| *TMEM115* | Transmembrane protein 115 | 429757 | 0.042 | 0.13 | 6.91 |
| *UBFD1* | Ubiquitin family domain containing 1 | 771251 | 0.043 | 0.13 | 4.92 |
| *VPS16* | VPS16 core subunit of CORVET and HOPS complexes | 426571 | 0.039 | 0.13 | 6.64 |
| *COIL* | Coilin | 417402 | 0.036 | 0.14 | 4.62 |
| *ELMO2* | Engulfment and cell motility 2 | 419307 | 0.036 | 0.14 | 5.98 |
| *EIF2S1* | Eukaryotic translation initiation factor 2 subunit alpha | 423279 | 0.031 | 0.14 | 6.91 |
| *FAM192A* | Family with sequence similarity 192 member A | 415647 | 0.02 | 0.14 | 6.01 |
| *GPN1* | GPN-loop GTPase 1 | 421980 | 0.011 | 0.14 | 5.27 |
| *HSPA4* | Heat shock protein family A (Hsp70) member 4 | 416339 | 0.033 | 0.14 | 8.08 |
| *LEO1* | LEO1 homolog, Paf1/RNA polymerase II complex component | 769405 | 0.034 | 0.14 | 4.24 |
| *MED27* | Mediator complex subunit 27 | 417172 | 0.048 | 0.14 | 4.47 |
| *MCRS1* | Microspherule protein 1 | 426664 | 0.039 | 0.14 | 6.67 |
| *PPIG* | Peptidylprolyl isomerase G | 424162 | 0.014 | 0.14 | 5.92 |
| *PPP1R7* | Protein phosphatase 1 regulatory subunit 7 | 424844 | 0.042 | 0.14 | 6.08 |
| *RBMX2* | RNA binding motif protein, X-linked 2 | 769358 | 0.048 | 0.14 | 6.1 |
| *POLR2C* | RNA polymerase II subunit C | 415635 | 0.044 | 0.14 | 5.51 |
| *POLR2D* | RNA polymerase II subunit D | 424754 | 0.043 | 0.14 | 4.7 |
| *SARNP* | SAP domain containing ribonucleoprotein | 425058 | 0.013 | 0.14 | 7.27 |
| *SNRPC* | Small nuclear ribonucleoprotein polypeptide C | 419901 | 0.023 | 0.14 | 6.38 |
| *SNX27* | Sorting nexin family member 27 | 425666 | 0.032 | 0.14 | 6.43 |
| *TAF12* | TATA-box binding protein associated factor 12 | 419585 | 0.02 | 0.14 | 5.63 |
| *VPS4A* | Vacuolar protein sorting 4 homolog A | 425637 | 0.039 | 0.14 | 7.25 |
| *CDC37* | Cell division cycle 37 | 395430 | 0.047 | 0.15 | 7.63 |
| *CPSF3* | Cleavage and polyadenylation specific factor 3 | 421929 | 0.007 | 0.15 | 5.99 |
| *COPS7B* | COP9 signalosome subunit 7B | 424933 | 0.042 | 0.15 | 4.2 |
| *EIF3D* | Eukaryotic translation initiation factor 3 subunit D | 771356 | 0.046 | 0.15 | 7.41 |
| *FIP1L1* | Factor interacting with PAPOLA and CPSF1 | 422754 | 0.016 | 0.15 | 6.7 |
| *HNRNPAB* | Heterogeneous nuclear ribonucleoprotein A/B | 396268 | 0.018 | 0.15 | 9.01 |
| *MLST8* | MTOR associated protein, LST8 homolog | 416558 | 0.034 | 0.15 | 5.85 |
| *NUP133* | Nucleoporin 133 | 421535 | 0.036 | 0.15 | 5.02 |
| *PRPF19* | Pre-mRNA processing factor 19 | 430767 | 0.02 | 0.15 | 7.26 |
| *PRPF38A* | Pre-mRNA processing factor 38A | 771795 | 0.018 | 0.15 | 5.39 |
| *PSMC1* | Proteasome 26S subunit, ATPase 1 | 395804 | 0.016 | 0.15 | 7.53 |
| *PSMC3* | Proteasome 26S subunit, ATPase 3 | 423182 | 0.029 | 0.15 | 8.19 |
| *PRPF40A* | PRP40 pre-mRNA processing factor 40 homolog A (S. cerevisiae) | 373972 | 0.017 | 0.15 | 7.68 |
| *SNRPD3* | Small nuclear ribonucleoprotein D3 polypeptide | 416947 | 0.029 | 0.15 | 6.32 |
| *TAB1* | TGF-beta activated kinase 1 (MAP3K7) binding protein 1 | 418014 | 0.014 | 0.15 | 5.06 |
| *UTP14A* | UTP14A small subunit processome component | 422140 | 0.021 | 0.15 | 6.36 |
| *UTP6* | UTP6, small subunit processome component | 417405 | 0.023 | 0.15 | 5.11 |
| *ACTR10* | Actin related protein 10 | 423543 | 0.009 | 0.16 | 5.44 |
| *CACYBP* | Calcyclin binding protein | 424437 | 0.042 | 0.16 | 6.55 |
| *CSTF2* | Cleavage stimulation factor subunit 2 | 422258 | 0.006 | 0.16 | 5.55 |
| *LOC112533016* | Collagen alpha-1(I) chain-like | 112533016 | 0.039 | 0.16 | 6.07 |
| *DAZAP1* | DAZ associated protein 1 | 427266 | 0.041 | 0.16 | 8.08 |
| *DHODH* | Dihydroorotate dehydrogenase (quinone) | 415876 | 0.028 | 0.16 | 5.06 |
| *EIF2B3* | Eukaryotic translation initiation factor 2B subunit gamma | 424587 | 0.021 | 0.16 | 4.76 |
| *GPALPP1* | GPALPP motifs containing 1 | 418842 | 0.02 | 0.16 | 4.92 |
| *HNRNPA2B1* | Heterogeneous nuclear ribonucleoprotein A2/B1 | 420627 | 0.022 | 0.16 | 8.78 |
| *HMGB1* | High mobility group box 1 | 395724 | 0.047 | 0.16 | 7.82 |
| *MVB12A* | Multivesicular body subunit 12A | 426089 | 0.043 | 0.16 | 6.25 |
| *NMT1* | N-myristoyltransferase 1 | 419966 | 0.035 | 0.16 | 5.48 |
| *PPIE* | Peptidylprolyl isomerase E | 426061 | 0.04 | 0.16 | 5.4 |
| *RSG1* | REM2 and RAB like small GTPase 1 | 107054909 | 0.048 | 0.16 | 5.16 |
| *SCNM1* | Sodium channel modifier 1 | 425744 | 0.039 | 0.16 | 5.67 |
| *TIMM22* | Translocase of inner mitochondrial membrane 22 | 417594 | 0.044 | 0.16 | 3.98 |
| *TSR3* | TSR3 ribosome maturation factor | 416590 | 0.045 | 0.16 | 4.62 |
| *UBE2G2* | Ubiquitin conjugating enzyme E2 G2 | 424837 | 0.018 | 0.16 | 7.11 |
| *CPSF4* | Cleavage and polyadenylation specific factor 4 | 416494 | 0.034 | 0.17 | 5.2 |
| *ERI3* | ERI1 exoribonuclease family member 3 | 424579 | 0.025 | 0.17 | 4.97 |
| *HNRNPC* | Heterogeneous nuclear ribonucleoprotein C | 121108713 | 0.044 | 0.17 | 5.41 |
| *LMAN2* | Lectin, mannose binding 2 | 100859676 | 0.013 | 0.17 | 7.2 |
| *LRR1* | Leucine rich repeat protein 1 | 423565 | 0.043 | 0.17 | 3.7 |
| *NUBP1* | Nucleotide binding protein 1 | 416634 | 0.017 | 0.17 | 5.4 |
| *PPCS* | Phosphopantothenoylcysteine synthetase | 100859789 | 0.023 | 0.17 | 4.69 |
| *PDCD11* | Programmed cell death 11 | 423874 | 0.044 | 0.17 | 4.74 |
| *SPCS3* | Signal peptidase complex subunit 3 | 396234 | 0.014 | 0.17 | 6.09 |
| *SRP72* | Signal recognition particle 72 | 422626 | 0.012 | 0.17 | 6.49 |
| *U2AF1* | U2 small nuclear RNA auxiliary factor 1 | 395370 | 0.005 | 0.17 | 7.07 |
| *VASP* | Vasodilator-stimulated phosphoprotein | 107057642 | 0.05 | 0.17 | 6.22 |
| *WDYHV1* | WDYHV motif containing 1 | 420345 | 0.034 | 0.17 | 3.01 |
| *RARS* | Arginyl-tRNA synthetase | 416168 | 0.006 | 0.18 | 6.2 |
| *ATG3* | Autophagy related 3 | 418369 | 0.023 | 0.18 | 5.45 |
| *CHCHD5* | Coiled-coil-helix-coiled-coil-helix domain containing 5 | 770886 | 0.025 | 0.18 | 4.8 |
| *ECHDC2* | Enoyl-CoA hydratase domain containing 2 | 424646 | 0.032 | 0.18 | 5.28 |
| *EEF1B2* | Eukaryotic translation elongation factor 1 beta 2 | 395723 | 0.038 | 0.18 | 8.95 |
| *EIF4EBP1* | Eukaryotic translation initiation factor 4E binding protein 1 | 426773 | 0.014 | 0.18 | 6.99 |
| *EARS2* | Glutamyl-tRNA synthetase 2, mitochondrial | 427672 | 0.034 | 0.18 | 2.81 |
| *MOB3A* | MOB kinase activator 3A | 100857182 | 0.028 | 0.18 | 6.85 |
| *MSH2* | MutS homolog 2 | 378799 | 0.026 | 0.18 | 5.01 |
| *NAA20* | N(alpha)-acetyltransferase 20, NatB catalytic subunit | 424333 | 0.017 | 0.18 | 5.95 |
| *NSUN2* | NOP2/Sun RNA methyltransferase family member 2 | 420938 | 0.04 | 0.18 | 4.81 |
| *NUP35* | Nucleoporin 35 | 121106451 | 0.028 | 0.18 | 3.77 |
| *NUP62* | Nucleoporin 62 | 422183 | 0.012 | 0.18 | 6.11 |
| *NUP93* | Nucleoporin 93 | 415693 | 0.041 | 0.18 | 4.64 |
| *PPID* | Peptidylprolyl isomerase D | 428725 | 0.023 | 0.18 | 5.18 |
| *PPIL4* | Peptidylprolyl isomerase like 4 | 421625 | 0.023 | 0.18 | 4.99 |
| *PHB2* | Prohibitin 2 | 771124 | 0.024 | 0.18 | 7.56 |
| *PSMA3* | Proteasome subunit alpha 3 | 423542 | 0.03 | 0.18 | 6.96 |
| *PUS3* | Pseudouridylate synthase 3 | 419708 | 0.032 | 0.18 | 4.22 |
| *RPL3* | Ribosomal protein L3 | 418016 | 0.032 | 0.18 | 9.57 |
| *RPL34* | Ribosomal protein L34 | 422526 | 0.02 | 0.18 | 8.87 |
| *POLR2H* | RNA polymerase II subunit H | 424954 | 0.035 | 0.18 | 4.81 |
| *SF3A3* | Splicing factor 3a subunit 3 | 419608 | 0.007 | 0.18 | 6.16 |
| *STT3A* | STT3A, catalytic subunit of the oligosaccharyltransferase complex | 100857165 | 0.013 | 0.18 | 7.57 |
| *TFAM* | Transcription factor A, mitochondrial | 373888 | 0.022 | 0.18 | 5.38 |
| *TIMM9* | Translocase of inner mitochondrial membrane 9 | 100859762 | 0.045 | 0.18 | 4.27 |
| *TRNT1* | TRNA nucleotidyl transferase 1 | 416103 | 0.017 | 0.18 | 5.35 |
| *COQ7* | Coenzyme Q7, hydroxylase | 416609 | 0.02 | 0.19 | 4 |
| *COMMD7* | COMM domain containing 7 | 419286 | 0.032 | 0.19 | 5.14 |
| *COX17* | COX17, cytochrome c oxidase copper chaperone | 770190 | 0.024 | 0.19 | 5.96 |
| *CTPS1* | CTP synthase 1 | 419561 | 0.049 | 0.19 | 5.09 |
| *DKC1* | Dyskerin pseudouridine synthase 1 | 422196 | 0.018 | 0.19 | 6.16 |
| *EIF3K* | Eukaryotic translation initiation factor 3 subunit K | 101750374 | 0.039 | 0.19 | 7.97 |
| *GTF2F2* | General transcription factor IIF subunit 2 | 418843 | 0.04 | 0.19 | 5.02 |
| *GTPBP4* | GTP binding protein 4 | 420458 | 0.005 | 0.19 | 5.94 |
| *HNRNPA1* | Heterogeneous nuclear ribonucleoprotein A1 | 424136 | 0.033 | 0.19 | 8.11 |
| *HTRA2* | HtrA serine peptidase 2 | 395990 | 0.03 | 0.19 | 6.28 |
| *IMPDH2* | Inosine monophosphate dehydrogenase 2 | 416058 | 0.024 | 0.19 | 6.49 |
| *INTS5* | Integrator complex subunit 5 | 101750875 | 0.037 | 0.19 | 6.43 |
| *LSM7* | LSM7 homolog, U6 small nuclear RNA and mRNA degradation associated | 420055 | 0.044 | 0.19 | 4.94 |
| *MED19* | Mediator complex subunit 19 | 428854 | 0.033 | 0.19 | 5.24 |
| *MALSU1* | Mitochondrial assembly of ribosomal large subunit 1 | 420616 | 0.01 | 0.19 | 5.21 |
| *MPC2* | Mitochondrial pyruvate carrier 2 | 768451 | 0.04 | 0.19 | 6.05 |
| *MRPL35* | Mitochondrial ribosomal protein L35 | 422914 | 0.027 | 0.19 | 5.36 |
| *MRPL46* | Mitochondrial ribosomal protein L46 | 415499 | 0.024 | 0.19 | 4.49 |
| *NIPSNAP2* | Nipsnap homolog 2 | 417539 | 0.013 | 0.19 | 6.12 |
| *NUTF2* | Nuclear transport factor 2 | 415655 | 0.022 | 0.19 | 5.77 |
| *NUP88* | Nucleoporin 88 | 417501 | 0.005 | 0.19 | 5.99 |
| *PSMB7* | Proteasome subunit beta 7 | 378915 | 0.018 | 0.19 | 7.47 |
| *PPP1CA* | Protein phosphatase 1 catalytic subunit alpha | 100858156 | 0.009 | 0.19 | 7.34 |
| *RABEPK* | Rab9 effector protein with kelch motifs | 772357 | 0.04 | 0.19 | 4.61 |
| *RPL6* | Ribosomal protein L6 | 373957 | 0.022 | 0.19 | 9.25 |
| *RRP7A* | Ribosomal RNA processing 7 homolog A | 417975 | 0.013 | 0.19 | 4.96 |
| *RPEL1* | Ribulose-5-phosphate-3-epimerase like 1 | 770045 | 0.009 | 0.19 | 6.29 |
| *RIOK1* | RIO kinase 1 | 420870 | 0.008 | 0.19 | 5.72 |
| *RBM48* | RNA binding motif protein 48 | 420555 | 0.035 | 0.19 | 4.32 |
| *RBMXL3* | RNA binding motif protein, X-linked like 3 | 416302 | 0.013 | 0.19 | 5.69 |
| *POLR2I* | RNA polymerase II subunit I | 426289 | 0.02 | 0.19 | 4.99 |
| *SLC13A3* | Solute carrier family 13 member 3 | 770495 | 0.04 | 0.19 | 3.63 |
| *SF3B4* | Splicing factor 3b subunit 4 | 426042 | 0.014 | 0.19 | 7.75 |
| *SERP1* | Stress associated endoplasmic reticulum protein 1 | 107054146 | 0.018 | 0.19 | 7.19 |
| *SMARCE1* | SWI/SNF related, matrix associated, actin dependent regulator of chromatin, subfamily e, member 1 | 420047 | 0.009 | 0.19 | 6.88 |
| *THOC1* | THO complex 1 | 421062 | 0.005 | 0.19 | 5.61 |
| *TBL3* | Transducin beta like 3 | 416545 | 0.009 | 0.19 | 5.42 |
| *UQCRC2* | Ubiquinol-cytochrome c reductase core protein II | 427009 | 0.04 | 0.19 | 7.36 |
| *USF1* | Upstream transcription factor 1 | 425126 | 0.009 | 0.19 | 6.93 |
| *VTI1B* | Vesicle transport through interaction with t-SNAREs 1B | 423273 | 0.02 | 0.19 | 5.02 |
| *WDR75* | WD repeat domain 75 | 423985 | 0.013 | 0.19 | 5.25 |
| *ANKRD61* | Ankyrin repeat domain 61 | 101752127 | 0.009 | 0.2 | 5.11 |
| *BBIP1* | BBSome interacting protein 1 | 100858359 | 0.032 | 0.2 | 3.82 |
| *B4GALT7* | Beta-1,4-galactosyltransferase 7 | 416359 | 0.013 | 0.2 | 4.64 |
| *CDK5RAP3* | CDK5 regulatory subunit associated protein 3 | 430350 | 0.016 | 0.2 | 5.65 |
| *CCT3* | Chaperonin containing TCP1 subunit 3 | 425644 | 0.019 | 0.2 | 7.75 |
| *CCT4* | Chaperonin containing TCP1 subunit 4 | 395414 | 0.035 | 0.2 | 8.13 |
| *DENR* | Density regulated re-initiation and release factor | 771794 | 0.005 | 0.2 | 5.1 |
| *DNTTIP1* | Deoxynucleotidyltransferase terminal interacting protein 1 | 431225 | 0.028 | 0.2 | 6.51 |
| *DRG1* | Developmentally regulated GTP binding protein 1 | 416962 | 0.005 | 0.2 | 5.24 |
| *DNAJC17* | DnaJ heat shock protein family (Hsp40) member C17 | 770519 | 0.035 | 0.2 | 4.02 |
| *DNAJC2* | DnaJ heat shock protein family (Hsp40) member C2 | 417717 | 0.029 | 0.2 | 6.52 |
| *DBNL* | Drebrin like | 425994 | 0.031 | 0.2 | 6.59 |
| *EBNA1BP2* | EBNA1 binding protein 2 | 424558 | 0.037 | 0.2 | 5.17 |
| *ERG28* | Ergosterol biosynthesis 28 | 423366 | 0.039 | 0.2 | 5.53 |
| *EIF3G* | Eukaryotic translation initiation factor 3, subunit G | 101747413 | 0.024 | 0.2 | 7.69 |
| *GNL2* | G protein nucleolar 2 | 419614 | 0.011 | 0.2 | 5.57 |
| *HM13* | Histocompatibility minor 13 | 100859573 | 0.017 | 0.2 | 7.02 |
| *LRRC42* | Leucine rich repeat containing 42 | 424655 | 0.01 | 0.2 | 4.34 |
| *MTHFD1* | Methylenetetrahydrofolate dehydrogenase, cyclohydrolase and formyltetrahydrofolate synthetase 1 | 423508 | 0.048 | 0.2 | 4.66 |
| *MRPL39* | Mitochondrial ribosomal protein L39 | 418475 | 0.019 | 0.2 | 4.16 |
| *NPM1* | Nucleophosmin | 396203 | 0.043 | 0.2 | 8.45 |
| *OTUB1* | OTU deubiquitinase, ubiquitin aldehyde binding 1 | 777320 | 0.02 | 0.2 | 5.67 |
| *POLR2J* | Polymerase (RNA) II (DNA directed) polypeptide J, 13.3kDa | 107049063 | 0.041 | 0.2 | 3.86 |
| *PSMC5* | Proteasome 26S subunit, ATPase 5 | 428274 | 0.013 | 0.2 | 7.09 |
| *PSMD11* | Proteasome 26S subunit, non-ATPase 11 | 430878 | 0.013 | 0.2 | 6.9 |
| *RAN* | RAN, member RAS oncogene family | 396193 | 0.032 | 0.2 | 7.42 |
| *RPL18A* | Ribosomal protein L18a | 417823 | 0.023 | 0.2 | 8.67 |
| *RPL24* | Ribosomal protein L24 | 418401 | 0.02 | 0.2 | 8.41 |
| *RPF2* | Ribosome production factor 2 homolog | 428624 | 0.029 | 0.2 | 4.89 |
| *SELENOK* | Selenoprotein K | 415995 | 0.035 | 0.2 | 5.4 |
| *SARS1* | Seryl-tRNA synthetase 1 | 426697 | 0.032 | 0.2 | 6.23 |
| *SIGMAR1* | Sigma non-opioid intracellular receptor 1 | 100859748 | 0.014 | 0.2 | 4.73 |
| *LOC420480* | Sjogren's syndrome nuclear autoantigen 1-like | 420480 | 0.024 | 0.2 | 4.19 |
| *SNRNP27* | Small nuclear ribonucleoprotein U4/U6.U5 subunit 27 | 771295 | 0.022 | 0.2 | 6.88 |
| *STOML2* | Stomatin like 2 | 427412 | 0.006 | 0.2 | 6.16 |
| *SSRP1* | Structure specific recognition protein 1 | 396509 | 0.018 | 0.2 | 7.6 |
| *TRIP4* | Thyroid hormone receptor interactor 4 | 415330 | 0.005 | 0.2 | 4.34 |
| *TBL2* | Transducin (beta)-like 2 | 101751077 | 0.043 | 0.2 | 6.32 |
| *TOMM20* | Translocase of outer mitochondrial membrane 20 | 426309 | 0.014 | 0.2 | 6.96 |
| *TRMT10A* | TRNA methyltransferase 10A | 422708 | 0.019 | 0.2 | 3.2 |
| *WDR12* | WD repeat domain 12 | 424089 | 0.008 | 0.2 | 5.31 |
| *AIMP1* | Aminoacyl tRNA synthetase complex interacting multifunctional protein 1 | 422533 | 0.006 | 0.21 | 5.27 |
| *LOC107050649* | AP-1 complex subunit sigma-1A-like | 107050649 | 0.032 | 0.21 | 5.62 |
| *BRIX1* | BRX1, biogenesis of ribosomes | 427433 | 0.026 | 0.21 | 5.61 |
| *CTNNBL1* | Catenin beta like 1 | 419299 | 0.004 | 0.21 | 5.78 |
| *CCT6A* | Chaperonin containing TCP1 subunit 6A | 417541 | 0.005 | 0.21 | 8.26 |
| *CCT8* | Chaperonin containing TCP1 subunit 8 | 418486 | 0.029 | 0.21 | 6.84 |
| *COPZ1* | Coatomer protein complex subunit zeta 1 | 107055416 | 0.048 | 0.21 | 5.57 |
| *CHCHD7* | Coiled-coil-helix-coiled-coil-helix domain containing 7 | 770709 | 0.026 | 0.21 | 4.78 |
| *CCDC97* | Coiled-coil domain containing 97 | 101748852 | 0.009 | 0.21 | 5.93 |
| *CWC15* | CWC15 spliceosome associated protein homolog | 418996 | 0.008 | 0.21 | 7 |
| *FAM110A* | Family with sequence similarity 110 member A | 100858650 | 0.029 | 0.21 | 3.38 |
| *HMGN4* | High mobility group nucleosomal binding domain 4 | 768675 | 0.037 | 0.21 | 8.36 |
| *HMGN5* | High mobility group nucleosome binding domain 5 | 422278 | 0.046 | 0.21 | 6.58 |
| *KPNB1* | Karyopherin subunit beta 1 | 426499 | 0.015 | 0.21 | 7.17 |
| *LYSMD1* | LysM domain containing 1 | 100857442 | 0.032 | 0.21 | 3.61 |
| *MED28* | Mediator complex subunit 28 | 425350 | 0.004 | 0.21 | 4.95 |
| *MRPL12* | Mitochondrial ribosomal protein L12 | 769031 | 0.042 | 0.21 | 6.7 |
| *MRPL21* | Mitochondrial ribosomal protein L21 | 423000 | 0.01 | 0.21 | 4.91 |
| *MRPL42* | Mitochondrial ribosomal protein L42 | 417899 | 0.017 | 0.21 | 5.19 |
| *MRPL44* | Mitochondrial ribosomal protein L44 | 424795 | 0.005 | 0.21 | 5.54 |
| *MSH6* | MutS homolog 6 | 421291 | 0.039 | 0.21 | 4.98 |
| *NDUFB5* | NADH:ubiquinone oxidoreductase subunit B5 | 424978 | 0.025 | 0.21 | 6.43 |
| *NOC2L* | NOC2 like nucleolar associated transcriptional repressor | 419433 | 0.005 | 0.21 | 5.63 |
| *NOP58* | NOP58 ribonucleoprotein | 424087 | 0.019 | 0.21 | 6.02 |
| *LOC423693* | Nucleolar RNA helicase 2-like | 423693 | 0.027 | 0.21 | 5.78 |
| *NUDT21* | Nudix hydrolase 21 | 100858636 | 0.005 | 0.21 | 5.63 |
| *PPIH* | Peptidylprolyl isomerase H | 419507 | 0.004 | 0.21 | 6.13 |
| *PHPT1* | Phosphohistidine phosphatase 1 | 770750 | 0.01 | 0.21 | 4.68 |
| *PHKG1* | Phosphorylase kinase catalytic subunit gamma 1 | 417543 | 0.005 | 0.21 | 6.19 |
| *PUF60* | Poly(U) binding splicing factor 60 | 426402 | 0.004 | 0.21 | 6.97 |
| *PFDN1* | Prefoldin subunit 1 | 416142 | 0.032 | 0.21 | 5.42 |
| *PTGES3* | Prostaglandin E synthase 3 | 100859133 | 0.01 | 0.21 | 8.6 |
| *PSMA5* | Proteasome subunit alpha 5 | 426937 | 0.019 | 0.21 | 6.75 |
| *RPL5* | Ribosomal protein L5 | 395269 | 0.015 | 0.21 | 9.55 |
| *RPS14* | Ribosomal protein S14 | 416275 | 0.012 | 0.21 | 8.58 |
| *RPS29* | Ribosomal protein S29 | 776054 | 0.034 | 0.21 | 8.59 |
| *SELENBP1* | Selenium binding protein 1 | 425664 | 0.037 | 0.21 | 6.59 |
| *SIVA1* | SIVA1 apoptosis inducing factor | 423493 | 0.011 | 0.21 | 3.62 |
| *SRRD* | SRR1 domain containing | 416908 | 0.042 | 0.21 | 5.33 |
| *STIP1* | Stress induced phosphoprotein 1 | 101748085 | 0.023 | 0.21 | 7.81 |
| *UTP11* | UTP11, small subunit processome component homolog | 419606 | 0.01 | 0.21 | 5.39 |
| *BABAM1* | BRISC and BRCA1 A complex member 1 | 776677 | 0.014 | 0.22 | 5.48 |
| *C12orf57* | C12orf57 homolog | 771099 | 0.047 | 0.22 | 5.68 |
| *CCT2* | Chaperonin containing TCP1 subunit 2 | 417846 | 0.026 | 0.22 | 7.03 |
| *COMMD3* | COMM domain containing 3 | 420507 | 0.047 | 0.22 | 4.06 |
| *COA6* | Cytochrome c oxidase assembly factor 6 | 771652 | 0.016 | 0.22 | 4.28 |
| *COX4I1* | Cytochrome c oxidase subunit 4I1 | 415826 | 0.028 | 0.22 | 8.34 |
| *DDX54* | DEAD-box helicase 54 | 416992 | 0.026 | 0.22 | 5.91 |
| *EIF5A2* | Eukaryotic translation initiation factor 5A2 | 396545 | 0.008 | 0.22 | 7.09 |
| *EXOSC9* | Exosome component 9 | 619530 | 0.013 | 0.22 | 5.64 |
| *FKBP3* | FK506 binding protein 3 | 395353 | 0.005 | 0.22 | 6.39 |
| *GPKOW* | G-patch domain and KOW motifs | 107050739 | 0.017 | 0.22 | 4.21 |
| *MAK16* | MAK16 homolog | 426924 | 0.009 | 0.22 | 4.95 |
| *METTL13* | Methyltransferase like 13 | 100857481 | 0.032 | 0.22 | 3.95 |
| *MRPL16* | Mitochondrial ribosomal protein L16 | 428840 | 0.012 | 0.22 | 5.44 |
| *MRPL27* | Mitochondrial ribosomal protein L27 | 422105 | 0.028 | 0.22 | 5.26 |
| *NAT10* | N-acetyltransferase 10 | 426609 | 0.02 | 0.22 | 4.58 |
| *NDUFA6* | NADH:ubiquinone oxidoreductase subunit A6 | 427897 | 0.014 | 0.22 | 5.17 |
| *NOL7* | Nucleolar protein 7 | 420835 | 0.028 | 0.22 | 5.3 |
| *PPIL3* | Peptidylprolyl isomerase like 3 | 424075 | 0.004 | 0.22 | 4.85 |
| *PPRC1* | Peroxisome proliferator-activated receptor gamma, coactivator-related 1 | 423848 | 0.039 | 0.22 | 4.85 |
| *PRPF4* | Pre-mRNA processing factor 4 | 417270 | 0.004 | 0.22 | 6.18 |
| *PSMD7* | Proteasome 26S subunit, non-ATPase 7 | 415869 | 0.006 | 0.22 | 7.07 |
| *GATD3AL1* | Putative glutamine amidotransferase like class 1 domain containing 3A-like1 | 418811 | 0.023 | 0.22 | 5.29 |
| *RPL4* | Ribosomal protein L4 | 415551 | 0.013 | 0.22 | 9.96 |
| *RPS2* | Ribosomal protein S2 | 416544 | 0.043 | 0.22 | 8.87 |
| *RPS6* | Ribosomal protein S6 | 396148 | 0.036 | 0.22 | 9.8 |
| *SURF6* | Surfeit 6 | 417160 | 0.03 | 0.22 | 5.18 |
| *TKTL1* | Transketolase like 1 | 415991 | 0.02 | 0.22 | 7.38 |
| *TMUB1* | Transmembrane and ubiquitin like domain containing 1 | 431107 | 0.022 | 0.22 | 6.31 |
| *UCK2* | Uridine-cytidine kinase 2 | 424406 | 0.031 | 0.22 | 4.87 |
| *WDR76* | WD repeat domain 76 | 426208 | 0.048 | 0.22 | 3.72 |
| *ATP5B* | ATP synthase, H+ transporting, mitochondrial F1 complex, beta polypeptide | 426673 | 0.026 | 0.23 | 9.61 |
| *CCT5* | Chaperonin containing TCP1 subunit 5 | 420930 | 0.013 | 0.23 | 7.55 |
| *COQ9* | Coenzyme Q9 | 415636 | 0.033 | 0.23 | 4.94 |
| *CHCHD1* | Coiled-coil-helix-coiled-coil-helix domain containing 1 | 423741 | 0.007 | 0.23 | 4.8 |
| *DAP3* | Death associated protein 3 | 425065 | 0.007 | 0.23 | 5.77 |
| *DNAJA1* | DnaJ heat shock protein family (Hsp40) member A1 | 427376 | 0.029 | 0.23 | 8.35 |
| *EIF2B1* | Eukaryotic translation initiation factor 2B subunit alpha | 100857736 | 0.007 | 0.23 | 3.84 |
| *EXOSC8* | Exosome component 8 | 100858560 | 0.01 | 0.23 | 4.79 |
| *GLTP* | Glycolipid transfer protein | 429534 | 0.011 | 0.23 | 4.15 |
| *HSCB* | HscB mitochondrial iron-sulfur cluster cochaperone | 768938 | 0.02 | 0.23 | 4.36 |
| *HSD17B10* | Hydroxysteroid (17-beta) dehydrogenase 10 | 425758 | 0.039 | 0.23 | 5.82 |
| *ITPA* | Inosine triphosphatase | 424390 | 0.044 | 0.23 | 3.8 |
| *MED30* | Mediator complex subunit 30 | 420284 | 0.01 | 0.23 | 4.07 |
| *MINOS1* | Mitochondrial inner membrane organizing system 1 | 419474 | 0.012 | 0.23 | 6.06 |
| *MRPL33* | Mitochondrial ribosomal protein L33 | 771953 | 0.024 | 0.23 | 5.74 |
| *MRM3* | Mitochondrial rRNA methyltransferase 3 | 417620 | 0.008 | 0.23 | 4.52 |
| *NDUFS3* | NADH:ubiquinone oxidoreductase core subunit S3 | 423179 | 0.008 | 0.23 | 6.36 |
| *NDUFA12* | NADH:ubiquinone oxidoreductase subunit A12 | 417907 | 0.016 | 0.23 | 5.41 |
| *NDUFS6* | NADH:ubiquinone oxidoreductase subunit S6 | 420976 | 0.023 | 0.23 | 5.45 |
| *NDUFV3* | NADH:ubiquinone oxidoreductase subunit V3 | 418541 | 0.009 | 0.23 | 6.07 |
| *NFKBIB* | NFKB inhibitor beta | 100858419 | 0.013 | 0.23 | 6.14 |
| *NCAPD2* | Non-SMC condensin I complex subunit D2 | 418275 | 0.043 | 0.23 | 5.34 |
| *NUDCD3* | NudC domain containing 3 | 770002 | 0.006 | 0.23 | 5.79 |
| *PNO1* | Partner of NOB1 homolog | 769547 | 0.009 | 0.23 | 4.34 |
| *PDCD2* | Programmed cell death 2 | 421550 | 0.008 | 0.23 | 3.98 |
| *PSMD4* | Proteasome 26S subunit, non-ATPase 4 | 100216364 | 0.01 | 0.23 | 6.8 |
| *PSMB1* | Proteasome subunit beta 1 | 421551 | 0.009 | 0.23 | 7.14 |
| *ROMO1* | Reactive oxygen species modulator 1 | 768833 | 0.028 | 0.23 | 5.89 |
| *RPL10A* | Ribosomal protein L10a | 419895 | 0.013 | 0.23 | 8.55 |
| *RPL12* | Ribosomal protein L12 | 417264 | 0.014 | 0.23 | 8.71 |
| *RPL15* | Ribosomal protein L15 | 428442 | 0.022 | 0.23 | 8.85 |
| *RPL7* | Ribosomal protein L7 | 420182 | 0.013 | 0.23 | 9.24 |
| *RRP15* | Ribosomal RNA processing 15 homolog | 421353 | 0.005 | 0.23 | 3.64 |
| *RBX1* | Ring-box 1 | 418001 | 0.008 | 0.23 | 5.6 |
| *SPCS2* | Signal peptidase complex subunit 2 | 419056 | 0.006 | 0.23 | 6.43 |
| *SNF8* | SNF8, ESCRT-II complex subunit | 419990 | 0.005 | 0.23 | 4.61 |
| *SF3A2* | Splicing factor 3a subunit 2 | 420077 | 0.006 | 0.23 | 6.24 |
| *THOC3* | THO complex 3 | 416232 | 0.004 | 0.23 | 5.38 |
| *TRAPPC2L* | Trafficking protein particle complex 2 like | 415847 | 0.029 | 0.23 | 4.7 |
| *UFC1* | Ubiquitin-fold modifier conjugating enzyme 1 | 107057454 | 0.006 | 0.23 | 4.7 |
| *UBL5* | Ubiquitin like 5 | 107057622 | 0.015 | 0.23 | 7.43 |
| *USMG5* | Up-regulated during skeletal muscle growth 5 homolog (mouse) | 423873 | 0.008 | 0.23 | 6.05 |
| *UROD* | Uroporphyrinogen decarboxylase | 424590 | 0.008 | 0.23 | 4.53 |
| *BOP1* | Block of proliferation 1 | 425985 | 0.018 | 0.24 | 6.02 |
| *BYSL* | Bystin like | 419927 | 0.004 | 0.24 | 5.3 |
| *C12orf65* | C12orf65 homolog | 416827 | 0.023 | 0.24 | 4.06 |
| *DPH2* | DPH2 homolog | 424574 | 0.019 | 0.24 | 2.47 |
| *GRSF1* | G-rich RNA sequence binding factor 1 | 422646 | 0.005 | 0.24 | 5.81 |
| *HAX1* | HCLS1 associated protein X-1 | 107055138 | 0.021 | 0.24 | 6.44 |
| *HYI* | Hydroxypyruvate isomerase (putative) | 424567 | 0.039 | 0.24 | 3.77 |
| *IFT43* | Intraflagellar transport 43 | 771922 | 0.033 | 0.24 | 1.73 |
| *JAGN1* | Jagunal homolog 1 | 107054427 | 0.014 | 0.24 | 4.17 |
| *MED29* | Mediator complex subunit 29 | 107055391 | 0.043 | 0.24 | 5.58 |
| *MRPL2* | Mitochondrial ribosomal protein L2 | 421256 | 0.008 | 0.24 | 6.46 |
| *MRPL23* | Mitochondrial ribosomal protein L23 | 374255 | 0.005 | 0.24 | 5.58 |
| *MRPL32* | Mitochondrial ribosomal protein L32 | 420773 | 0.005 | 0.24 | 3.96 |
| *MRPS25* | Mitochondrial ribosomal protein S25 | 416129 | 0.005 | 0.24 | 5.83 |
| *NDUFA8* | NADH:ubiquinone oxidoreductase subunit A8 | 417112 | 0.009 | 0.24 | 5.85 |
| *PDRG1* | P53 and DNA damage regulated 1 | 771852 | 0.022 | 0.24 | 4.99 |
| *PPIB* | Peptidylprolyl isomerase B (cyclophilin B) | 396447 | 0.01 | 0.24 | 8.91 |
| *PFDN5* | Prefoldin subunit 5 | 100859376 | 0.013 | 0.24 | 6.91 |
| *PAM16* | Presequence translocase associated motor 16 homolog | 416669 | 0.015 | 0.24 | 5.55 |
| *PREB* | Prolactin regulatory element binding | 100858986 | 0.006 | 0.24 | 4.4 |
| *PA2G4* | Proliferation-associated 2G4, 38kDa | 425279 | 0.012 | 0.24 | 7.17 |
| *PSMA7* | Proteasome subunit alpha 7 | 395318 | 0.007 | 0.24 | 7.2 |
| *RSL1D1* | Ribosomal L1 domain containing 1 | 416416 | 0.011 | 0.24 | 5.32 |
| *RIOX1* | Ribosomal oxygenase 1 | 423249 | 0.004 | 0.24 | 4.37 |
| *RRP12* | Ribosomal RNA processing 12 homolog | 423845 | 0.017 | 0.24 | 5.36 |
| *SSBP1* | Single stranded DNA binding protein 1 | 418122 | 0.006 | 0.24 | 4.96 |
| *SLC25A5* | Solute carrier family 25 member 5 | 772225 | 0.018 | 0.24 | 7.05 |
| *TMEM141* | Transmembrane protein 141 | 417294 | 0.034 | 0.24 | 4.08 |
| *LOC768709* | Uncharacterized LOC768709 | 768709 | 0.028 | 0.24 | 4.63 |
| *UTP4* | UTP4, small subunit processome component | 769632 | 0.032 | 0.24 | 4.49 |
| *HPDL* | 4-hydroxyphenylpyruvate dioxygenase-like | 101748258 | 0.043 | 0.25 | 2.61 |
| *AHSA1* | Activator of Hsp90 ATPase activity 1 | 423378 | 0.005 | 0.25 | 7.1 |
| *ATP5H* | ATP synthase, H+ transporting, mitochondrial Fo complex subunit D | 422115 | 0.014 | 0.25 | 7.34 |
| *ATP5J* | ATP synthase, H+ transporting, mitochondrial Fo complex subunit F6 | 418477 | 0.023 | 0.25 | 6.77 |
| *BCAS2* | Breast carcinoma amplified sequence 2 | 419884 | 0.009 | 0.25 | 5.75 |
| *CLDN3* | Claudin 3 | 374029 | 0.05 | 0.25 | 7.36 |
| *COX7B* | Cytochrome c oxidase subunit 7B | 771947 | 0.014 | 0.25 | 7.63 |
| *DAD1* | Defender against cell death 1 | 395343 | 0.006 | 0.25 | 5.75 |
| *HDDC2* | HD domain containing 2 | 421719 | 0.011 | 0.25 | 4.29 |
| *HSP90B1* | Heat shock protein 90 beta family member 1 | 374163 | 0.02 | 0.25 | 9.21 |
| *IER3IP1* | Immediate early response 3 interacting protein 1 | 101749130 | 0.019 | 0.25 | 4.85 |
| *LIN37* | Lin-37 DREAM MuvB core complex component | 121108874 | 0.013 | 0.25 | 4.81 |
| *LSM3* | LSM3 homolog, U6 small nuclear RNA and mRNA degradation associated | 416040 | 0.014 | 0.25 | 4.42 |
| *MRPL20* | Mitochondrial ribosomal protein L20 | 770979 | 0.01 | 0.25 | 5.78 |
| *MRPS14* | Mitochondrial ribosomal protein S14 | 424436 | 0.007 | 0.25 | 5.11 |
| *NDUFV2* | NADH:ubiquinone oxidoreductase core subunit V2 | 426488 | 0.01 | 0.25 | 6.4 |
| *NDUFS4* | NADH:ubiquinone oxidoreductase subunit S4 | 374122 | 0.046 | 0.25 | 5.81 |
| *OXA1L* | OXA1L mitochondrial inner membrane protein | 112531359 | 0.009 | 0.25 | 5.51 |
| *PRELID1* | PRELI domain containing 1 | 395819 | 0.009 | 0.25 | 7.75 |
| *PSMC3IP* | PSMC3 interacting protein | 772119 | 0.048 | 0.25 | 4.7 |
| *RCC1* | Regulator of chromosome condensation 1 | 429810 | 0.019 | 0.25 | 4.96 |
| *RPL11* | Ribosomal protein L11 | 419682 | 0.007 | 0.25 | 8.86 |
| *RPL35* | Ribosomal protein L35 | 374133 | 0.008 | 0.25 | 8.24 |
| *RPS10* | Ribosomal protein S10 | 419904 | 0.016 | 0.25 | 9.25 |
| *RPS20* | Ribosomal protein S20 | 430990 | 0.031 | 0.25 | 8.73 |
| *RPSAP58* | Ribosomal protein SA pseudogene 58 | 395181 | 0.015 | 0.25 | 9.88 |
| *SEC11C* | SEC11 homolog C, signal peptidase complex subunit | 426850 | 0.005 | 0.25 | 5.64 |
| *TAF10* | TATA-box binding protein associated factor 10 | 107052460 | 0.013 | 0.25 | 5.17 |
| *TMA16* | Translation machinery associated 16 homolog | 770087 | 0.011 | 0.25 | 4.14 |
| *TOMM6* | Translocase of outer mitochondrial membrane 6 | 419924 | 0.007 | 0.25 | 5.58 |
| *TSEN15* | TRNA splicing endonuclease subunit 15 | 424448 | 0.021 | 0.25 | 2.8 |
| *UQCR10* | Ubiquinol-cytochrome c reductase, complex III subunit X | 770937 | 0.048 | 0.25 | 6.8 |
| *UQCR11* | Ubiquinol-cytochrome c reductase, complex III subunit XI | 770135 | 0.03 | 0.25 | 6.1 |
| *UBE2M* | Ubiquitin conjugating enzyme E2 M | 107049465 | 0.01 | 0.25 | 6.36 |
| *ARPC4* | Actin related protein 2/3 complex subunit 4 | 416051 | 0.012 | 0.26 | 7.3 |
| *BRICD5* | BRICHOS domain containing 5 | 107054618 | 0.028 | 0.26 | 4.37 |
| *CALR* | Calreticulin | 100859104 | 0.006 | 0.26 | 10.22 |
| *DNAJC9* | DnaJ heat shock protein family (Hsp40) member C9 | 423640 | 0.009 | 0.26 | 4.33 |
| *ELOF1* | Elongation factor 1 homolog | 107057620 | 0.008 | 0.26 | 4.4 |
| *FAM162A* | Family with sequence similarity 162 member A | 418270 | 0.01 | 0.26 | 4.48 |
| *GSG2* | Germ cell associated 2, haspin | 427834 | 0.048 | 0.26 | 2.99 |
| *H1FX* | H1 histone family member X | 107054440 | 0.023 | 0.26 | 6.56 |
| *KRT8* | Keratin 8 | 426896 | 0.02 | 0.26 | 6.98 |
| *MIF* | Macrophage migration inhibitory factor (glycosylation-inhibiting factor) | 100857237 | 0.016 | 0.26 | 6.21 |
| *NDUFC2* | NADH dehydrogenase (ubiquinone) 1, subcomplex unknown, 2, 14.5kDa | 404751 | 0.008 | 0.26 | 5.65 |
| *NDUFS8* | NADH:ubiquinone oxidoreductase core subunit S8 | 769492 | 0.01 | 0.26 | 6.7 |
| *NACA* | Nascent polypeptide-associated complex alpha subunit | 396544 | 0.017 | 0.26 | 8.46 |
| *NUDT16L1* | Nudix hydrolase 16 like 1 | 395557 | 0.006 | 0.26 | 6.88 |
| *PSMB4* | Proteasome subunit beta 4 | 429986 | 0.005 | 0.26 | 7.34 |
| *RPL21* | Ribosomal protein L21 | 418933 | 0.017 | 0.26 | 9.14 |
| *RPL8* | Ribosomal protein L8 | 418568 | 0.008 | 0.26 | 9.72 |
| *RPS16* | Ribosomal protein S16 | 417871 | 0.008 | 0.26 | 8.61 |
| *RPS4Y1* | Ribosomal protein S4, Y-linked 1 | 396001 | 0.01 | 0.26 | 9.59 |
| *SLC37A4* | Solute carrier family 37 member 4 | 419789 | 0.008 | 0.26 | 8.97 |
| *SF3B6* | Splicing factor 3b subunit 6 | 421976 | 0.005 | 0.26 | 5.13 |
| *UBB* | Ubiquitin B | 396190 | 0.014 | 0.26 | 8.91 |
| *YBX1* | Y-box binding protein 1 | 386575 | 0.008 | 0.26 | 9.51 |
| *ATP5G2* | ATP synthase, H+ transporting, mitochondrial Fo complex subunit C2 (subunit 9) | 100858216 | 0.014 | 0.27 | 6.44 |
| *C1orf131* | C1orf131 homolog | 421547 | 0.014 | 0.27 | 5.21 |
| *COMMD4* | COMM domain containing 4 | 770048 | 0.02 | 0.27 | 4.75 |
| *COX7A2* | Cytochrome c oxidase subunit 7A2 | 772260 | 0.015 | 0.27 | 6.97 |
| *GRHPR* | Glyoxylate and hydroxypyruvate reductase | 426806 | 0.04 | 0.27 | 4.2 |
| *HIGD2A* | HIG1 hypoxia inducible domain family member 2A | 416227 | 0.039 | 0.27 | 5.17 |
| *MOGS* | Mannosyl-oligosaccharide glucosidase | 107049491 | 0.026 | 0.27 | 5.23 |
| *MRPL19* | Mitochondrial ribosomal protein L19 | 422066 | 0.004 | 0.27 | 5.19 |
| *LOC121107538* | Mitochondrial ribosomal protein L9 | 121107538 | 0.01 | 0.27 | 6.39 |
| *MRPS11* | Mitochondrial ribosomal protein S11 | 415498 | 0.004 | 0.27 | 5.85 |
| *MRPS22* | Mitochondrial ribosomal protein S22 | 424824 | 0.006 | 0.27 | 4.98 |
| *MRTO4* | MRT4 homolog, ribosome maturation factor | 428192 | 0.017 | 0.27 | 6.55 |
| *NDUFB3* | NADH:ubiquinone oxidoreductase subunit B3 | 424078 | 0.011 | 0.27 | 6.17 |
| *NDUFB6* | NADH:ubiquinone oxidoreductase subunit B6 | 416391 | 0.033 | 0.27 | 6.67 |
| *NDUFS5* | NADH:ubiquinone oxidoreductase subunit S5 | 771510 | 0.022 | 0.27 | 6.42 |
| *NHP2* | NHP2 ribonucleoprotein | 416218 | 0.013 | 0.27 | 5.81 |
| *NUP42* | Nucleoporin 42 | 420614 | 0.004 | 0.27 | 5.07 |
| *FARSA* | Phenylalanyl-tRNA synthetase alpha subunit | 100859604 | 0.006 | 0.27 | 5.53 |
| *PRIM1* | Primase (DNA) subunit 1 | 426646 | 0.007 | 0.27 | 5.07 |
| *RPS15* | Ribosomal protein S15 | 396448 | 0.036 | 0.27 | 8.58 |
| *RPS15A* | Ribosomal protein S15a | 427675 | 0.031 | 0.27 | 9.05 |
| *RPS7* | Ribosomal protein S7 | 421919 | 0.032 | 0.27 | 8.84 |
| *RBM8A* | RNA binding motif protein 8A | 107050968 | 0.008 | 0.27 | 6.12 |
| *SNRPN* | Small nuclear ribonucleoprotein polypeptide N | 395298 | 0.006 | 0.27 | 6.09 |
| *TCP1* | T-complex 1 | 421586 | 0.005 | 0.27 | 7.7 |
| *TALDO1* | Transaldolase 1 | 423019 | 0.011 | 0.27 | 7.31 |
| *TIMM10B* | Translocase of inner mitochondrial membrane 10B | 107052461 | 0.01 | 0.27 | 3.38 |
| *TMEM223* | Transmembrane protein 223 | 107049451 | 0.011 | 0.27 | 4.91 |
| *TUBA5* | Tubulin, alpha 5 | 421169 | 0.044 | 0.27 | 6.07 |
| *ATP5G1* | ATP synthase, H+ transporting, mitochondrial Fo complex subunit C1 (subunit 9) | 419992 | 0.01 | 0.28 | 7.15 |
| *ATP5L* | ATP synthase, H+ transporting, mitochondrial Fo complex subunit G | 101749042 | 0.014 | 0.28 | 7.04 |
| *CDC25A* | Cell division cycle 25A | 420375 | 0.039 | 0.28 | 3.41 |
| *CENPS* | Centromere protein S | 771417 | 0.032 | 0.28 | 3.36 |
| *CBX5* | Chromobox 5 | 776802 | 0.042 | 0.28 | 4.65 |
| *C1QBP* | Complement C1q binding protein | 395538 | 0.027 | 0.28 | 6.59 |
| *LOC121108888* | Cytochrome c oxidase subunit 6B1 | 121108888 | 0.024 | 0.28 | 7.68 |
| *EXOSC7* | Exosome component 7 | 420704 | 0.004 | 0.28 | 4.68 |
| *FEN1* | Flap structure-specific endonuclease 1 | 769677 | 0.037 | 0.28 | 4.8 |
| *HSPE1* | Heat shock protein family E (Hsp10) member 1 | 395948 | 0.01 | 0.28 | 7.19 |
| *HDGF* | Hepatoma-derived growth factor | 776936 | 0.008 | 0.28 | 8.76 |
| *MRPL40* | Mitochondrial ribosomal protein L40 | 100858795 | 0.009 | 0.28 | 5.13 |
| *MRPL51* | Mitochondrial ribosomal protein L51 | 418274 | 0.004 | 0.28 | 5.54 |
| *MRPS21* | Mitochondrial ribosomal protein S21 | 426666 | 0.018 | 0.28 | 5.26 |
| *MRPS24* | Mitochondrial ribosomal protein S24 | 112530176 | 0.022 | 0.28 | 3.79 |
| *MYL6* | Myosin, light chain 6, alkali, smooth muscle and non-muscle | 100996929 | 0.039 | 0.28 | 9.83 |
| *NDUFS7* | NADH:ubiquinone oxidoreductase core subunit S7 | 770724 | 0.005 | 0.28 | 6.77 |
| *NDUFAB1* | NADH:ubiquinone oxidoreductase subunit AB1 | 416571 | 0.008 | 0.28 | 5.73 |
| *NDUFB11* | NADH:ubiquinone oxidoreductase subunit B11 | 121108932 | 0.01 | 0.28 | 6.11 |
| *NIP7* | NIP7, nucleolar pre-rRNA processing protein | 415863 | 0.021 | 0.28 | 4.38 |
| *PPIA* | Peptidylprolyl isomerase A | 776282 | 0.013 | 0.28 | 9.18 |
| *PBDC1* | Polysaccharide biosynthesis domain containing 1 | 100858203 | 0.039 | 0.28 | 5.84 |
| *RMI2* | RecQ mediated genome instability 2 | 416628 | 0.023 | 0.28 | 2.86 |
| *RPL17* | Ribosomal protein L17 | 426845 | 0.036 | 0.28 | 9.32 |
| *RPL19* | Ribosomal protein L19 | 420003 | 0.012 | 0.28 | 9.69 |
| *RPL30* | Ribosomal protein L30 | 425416 | 0.005 | 0.28 | 8.26 |
| *RPS12* | Ribosomal protein S12 | 421698 | 0.027 | 0.28 | 9.34 |
| *RPS24* | Ribosomal protein S24 | 423726 | 0.032 | 0.28 | 9.24 |
| *RPS3* | Ribosomal protein S3 | 419069 | 0.006 | 0.28 | 9.28 |
| *POLR1D* | RNA polymerase I subunit D | 428713 | 0.006 | 0.28 | 3.63 |
| *VPS72* | Vacuolar protein sorting 72 homolog | 425660 | 0.005 | 0.28 | 6.29 |
| *ATP5O* | ATP synthase, H+ transporting, mitochondrial F1 complex, O subunit | 418508 | 0.005 | 0.29 | 7.74 |
| *CNPY3* | Canopy FGF signaling regulator 3 | 100858990 | 0.006 | 0.29 | 6.7 |
| *EMG1* | EMG1, N1-specific pseudouridine methyltransferase | 418292 | 0.004 | 0.29 | 3.61 |
| *ESYT1* | Extended synaptotagmin 1 | 107055399 | 0.009 | 0.29 | 7.67 |
| *FKBP11* | FK506 binding protein 11 | 107049506 | 0.02 | 0.29 | 5.41 |
| *FAHD2A* | Fumarylacetoacetate hydrolase domain containing 2A | 426684 | 0.016 | 0.29 | 5.4 |
| *GADD45GIP1* | GADD45G interacting protein 1 | 107055382 | 0.004 | 0.29 | 5.9 |
| *GLTSCR2* | Glioma tumor suppressor candidate region gene 2 | 107049993 | 0.022 | 0.29 | 7.02 |
| *HMGB2* | High mobility group box 2 | 396482 | 0.046 | 0.29 | 5.79 |
| *INAFM2* | InaF motif containing 2 | 107053356 | 0.037 | 0.29 | 3 |
| *KNSTRN* | Kinetochore-localized astrin/SPAG5 binding protein | 426704 | 0.048 | 0.29 | 4.18 |
| *MRPL18* | Mitochondrial ribosomal protein L18 | 421585 | 0.005 | 0.29 | 5.99 |
| *MRPL24* | Mitochondrial ribosomal protein L24 | 768957 | 0.005 | 0.29 | 6.28 |
| *MRPL58* | Mitochondrial ribosomal protein L58 | 422114 | 0.005 | 0.29 | 5.08 |
| *MYCBP* | MYC binding protein | 770496 | 0.007 | 0.29 | 4.98 |
| *NDUFA1* | NADH:ubiquinone oxidoreductase subunit A1 | 772150 | 0.013 | 0.29 | 5.75 |
| *NDUFA13* | NADH:ubiquinone oxidoreductase subunit A13 | 100859320 | 0.012 | 0.29 | 6.44 |
| *NSMCE3* | NSE3 homolog, SMC5-SMC6 complex component | 100125831 | 0.029 | 0.29 | 5.18 |
| *ORC4* | Origin recognition complex subunit 4 | 424307 | 0.012 | 0.29 | 4.07 |
| *RPL27A* | Ribosomal protein L27a | 770018 | 0.009 | 0.29 | 8.71 |
| *RPL29* | Ribosomal protein L29 | 770249 | 0.009 | 0.29 | 8.32 |
| *RPL9* | Ribosomal protein L9 | 425468 | 0.008 | 0.29 | 9.36 |
| *RPS11* | Ribosomal protein S11 | 419049 | 0.009 | 0.29 | 9.19 |
| *RPS23* | Ribosomal protein S23 | 427323 | 0.017 | 0.29 | 9.1 |
| *RPS8* | Ribosomal protein S8 | 424584 | 0.008 | 0.29 | 9.74 |
| *POLR2F* | RNA polymerase II subunit F | 395486 | 0.005 | 0.29 | 6.2 |
| *SDCCAG3* | Serologically defined colon cancer antigen 3 | 772136 | 0.012 | 0.29 | 3.34 |
| *SNRPA1* | Small nuclear ribonucleoprotein A' | 415523 | 0.004 | 0.29 | 5.28 |
| *SNRPE* | Small nuclear ribonucleoprotein polypeptide E | 396180 | 0.011 | 0.29 | 5.63 |
| *SF3B5* | Splicing factor 3b subunit 5 | 769794 | 0.017 | 0.29 | 5.25 |
| *TARBP2* | TARBP2, RISC loading complex RNA binding subunit | 107055413 | 0.011 | 0.29 | 4.05 |
| *TMEM258* | Transmembrane protein 258 | 422964 | 0.011 | 0.29 | 5.61 |
| *TPD52* | Tumor protein D52 | 770339 | 0.031 | 0.29 | 3.71 |
| *UBA52* | Ubiquitin A-52 residue ribosomal protein fusion product 1 | 395958 | 0.011 | 0.29 | 8.54 |
| *ATRAID* | All-trans retinoic acid induced differentiation factor | 776812 | 0.02 | 0.3 | 4.67 |
| *ALYREF* | Aly/REF export factor | 769169 | 0.004 | 0.3 | 6.29 |
| *ECHDC1* | Ethylmalonyl-CoA decarboxylase 1 | 769021 | 0.029 | 0.3 | 1.64 |
| *EEF1G* | Eukaryotic translation elongation factor 1 gamma | 100526660 | 0.005 | 0.3 | 8.4 |
| *LAMTOR2* | Late endosomal/lysosomal adaptor, MAPK and MTOR activator 2 | 100859842 | 0.02 | 0.3 | 5.89 |
| *MCPH1* | Microcephalin 1 | 100125976 | 0.046 | 0.3 | 2.03 |
| *MCM5* | Minichromosome maintenance complex component 5 | 418058 | 0.011 | 0.3 | 5.63 |
| *MRPL17* | Mitochondrial ribosomal protein L17 | 769700 | 0.005 | 0.3 | 5.59 |
| *PTRHD1* | Peptidyl-tRNA hydrolase domain containing 1 | 422015 | 0.014 | 0.3 | 2.88 |
| *POC1A* | POC1 centriolar protein A | 415889 | 0.009 | 0.3 | 3.12 |
| *PSMB3* | Proteasome subunit beta 3 | 419997 | 0.004 | 0.3 | 6.09 |
| *PTPMT1* | Protein tyrosine phosphatase, mitochondrial 1 | 423181 | 0.016 | 0.3 | 4.56 |
| *RPL14* | Ribosomal protein L14 | 374134 | 0.009 | 0.3 | 8.47 |
| *RPL37* | Ribosomal protein L37 | 427186 | 0.008 | 0.3 | 8.75 |
| *RPL37A* | Ribosomal protein L37a | 769981 | 0.014 | 0.3 | 8.82 |
| *RPS13* | Ribosomal protein S13 | 414782 | 0.005 | 0.3 | 7.93 |
| *RPS21* | Ribosomal protein S21 | 419228 | 0.014 | 0.3 | 7.02 |
| *POLR2L* | RNA polymerase II subunit L | 770704 | 0.004 | 0.3 | 4.75 |
| *SSSCA1* | Sjogren syndrome/scleroderma autoantigen 1 | 112530946 | 0.033 | 0.3 | 3.17 |
| *SNRPD2* | Small nuclear ribonucleoprotein D2 polypeptide | 107049207 | 0.007 | 0.3 | 6.05 |
| *SKA3* | Spindle and kinetochore associated complex subunit 3 | 418948 | 0.036 | 0.3 | 3.78 |
| *C12orf10* | C12orf10 homolog | 426187 | 0.017 | 0.31 | 3.8 |
| *CDC26* | Cell division cycle 26 | 417269 | 0.014 | 0.31 | 4.99 |
| *CDT1* | Chromatin licensing and DNA replication factor 1 | 426254 | 0.027 | 0.31 | 5.08 |
| *CCNB1* | Cyclin B1 | 415400 | 0.033 | 0.31 | 5.55 |
| *CDK1* | Cyclin dependent kinase 1 | 396252 | 0.036 | 0.31 | 6.12 |
| *ELOB* | Elongin B | 121108722 | 0.006 | 0.31 | 5.6 |
| *EXOSC1* | Exosome component 1 | 423844 | 0.031 | 0.31 | 5.41 |
| *MCRIP2* | MAPK regulated corepressor interacting protein 2 | 425184 | 0.008 | 0.31 | 5.24 |
| *MRPL50* | Mitochondrial ribosomal protein L50 | 427307 | 0.004 | 0.31 | 4.9 |
| *MRPS10* | Mitochondrial ribosomal protein S10 | 421385 | 0.004 | 0.31 | 4.31 |
| *NME2* | NME/NM23 nucleoside diphosphate kinase 2 | 395916 | 0.005 | 0.31 | 8.11 |
| *PBK* | PDZ binding kinase | 422003 | 0.029 | 0.31 | 4.57 |
| *PSMB5* | Proteasome subunit beta 5 | 396003 | 0.004 | 0.31 | 7.48 |
| *RABIF* | RAB interacting factor | 421170 | 0.005 | 0.31 | 4.93 |
| *RAD18* | RAD18, E3 ubiquitin protein ligase | 416114 | 0.008 | 0.31 | 3.7 |
| *RPL31* | Ribosomal protein L31 | 418710 | 0.016 | 0.31 | 8.32 |
| *RPL32* | Ribosomal protein L32 | 416122 | 0.01 | 0.31 | 9.2 |
| *RPLP2* | Ribosomal protein lateral stalk subunit P2 | 426492 | 0.009 | 0.31 | 9.1 |
| *RPS26* | Ribosomal protein S26 | 100857770 | 0.013 | 0.31 | 8.71 |
| *RPS28* | Ribosomal protein S28 | 768930 | 0.006 | 0.31 | 7.66 |
| *SVBP* | Small vasohibin binding protein | 101749360 | 0.009 | 0.31 | 6.92 |
| *SPC25* | SPC25, NDC80 kinetochore complex component | 424172 | 0.038 | 0.31 | 4.11 |
| *ATP5E* | ATP synthase, H+ transporting, mitochondrial F1 complex, epsilon subunit | 768660 | 0.011 | 0.32 | 6.83 |
| *ATPIF1* | ATPase inhibitory factor 1 | 100857945 | 0.005 | 0.32 | 6.58 |
| *CHCHD10* | Coiled-coil-helix-coiled-coil-helix domain containing 10 | 416933 | 0.041 | 0.32 | 6.37 |
| *COX6A1* | Cytochrome c oxidase subunit 6A1 | 416978 | 0.013 | 0.32 | 7.4 |
| *HYPK* | Huntingtin interacting protein K | 770307 | 0.005 | 0.32 | 5.9 |
| *MRPS9* | Mitochondrial ribosomal protein S9 | 418723 | 0.005 | 0.32 | 5.27 |
| *NDUFAF3* | NADH:ubiquinone oxidoreductase complex assembly factor 3 | 107054424 | 0.024 | 0.32 | 4.87 |
| *NDUFA2* | NADH:ubiquinone oxidoreductase subunit A2 | 768860 | 0.005 | 0.32 | 5.2 |
| *NASP* | Nuclear autoantigenic sperm protein | 424600 | 0.005 | 0.32 | 5.91 |
| *RRM2* | Ribonucleotide reductase regulatory subunit M2 | 421936 | 0.014 | 0.32 | 5.56 |
| *RPL13* | Ribosomal protein L13 | 395849 | 0.005 | 0.32 | 9.15 |
| *RPL22L1* | Ribosomal protein L22 like 1 | 100858296 | 0.035 | 0.32 | 6.39 |
| *RPL26L1* | Ribosomal protein L26 like 1 | 396400 | 0.009 | 0.32 | 9 |
| *RPS17* | Ribosomal protein S17 | 374053 | 0.009 | 0.32 | 8.97 |
| *SRA1* | Steroid receptor RNA activator 1 | 427608 | 0.009 | 0.32 | 4.91 |
| *TRAIP* | TRAF interacting protein | 415927 | 0.046 | 0.32 | 3.09 |
| *UQCRQ* | Ubiquinol-cytochrome c reductase complex III subunit VII | 416336 | 0.007 | 0.32 | 7.32 |
| *ZFAND2A* | Zinc finger AN1-type containing 2A | 425104 | 0.048 | 0.32 | 6.78 |
| *ATP5D* | ATP synthase, H+ transporting, mitochondrial F1 complex, delta subunit | 771478 | 0.006 | 0.33 | 7.55 |
| *BOLA3* | BolA family member 3 | 107054980 | 0.022 | 0.33 | 4.58 |
| *CDC6* | Cell division cycle 6 | 100857489 | 0.018 | 0.33 | 3.87 |
| *CDCA3* | Cell division cycle associated 3 | 770517 | 0.022 | 0.33 | 4.03 |
| *CENPJ* | Centromere protein J | 418960 | 0.009 | 0.33 | 2.2 |
| *CENPM* | Centromere protein M | 417982 | 0.026 | 0.33 | 1.69 |
| *C19orf70* | Chromosome 19 open reading frame 70 | 100858091 | 0.005 | 0.33 | 4.45 |
| *C26H6ORF125* | Chromosome 26 open reading frame, human C6orf125 | 419909 | 0.005 | 0.33 | 5.07 |
| *EXOSC3* | Exosome component 3 | 423626 | 0.004 | 0.33 | 3.27 |
| *HAUS1* | HAUS augmin like complex subunit 1 | 416380 | 0.039 | 0.33 | 3.21 |
| *HDDC3* | HD domain containing 3 | 107056801 | 0.035 | 0.33 | 4.24 |
| *JTB* | Jumping translocation breakpoint | 770053 | 0.005 | 0.33 | 6.81 |
| *LOC101751218* | MAX dimerization protein 3 | 101751218 | 0.039 | 0.33 | 3.33 |
| *MANF* | Mesencephalic astrocyte derived neurotrophic factor | 107048996 | 0.005 | 0.33 | 6.5 |
| *MRPL43* | Mitochondrial ribosomal protein L43 | 100858689 | 0.007 | 0.33 | 4.71 |
| *NDUFA11* | NADH:ubiquinone oxidoreductase subunit A11 | 420060 | 0.005 | 0.33 | 5.61 |
| *PTTG2* | Pituitary tumor-transforming 2 | 416158 | 0.044 | 0.33 | 4.46 |
| *LOC107050614* | Prefoldin subunit 6-like | 107050614 | 0.013 | 0.33 | 6.07 |
| *RPL22* | Ribosomal protein L22 | 373937 | 0.013 | 0.33 | 8.33 |
| *RPL27* | Ribosomal protein L27 | 396280 | 0.008 | 0.33 | 9.03 |
| *RPL36* | Ribosomal protein L36 | 373936 | 0.008 | 0.33 | 7.72 |
| *RPL36A* | Ribosomal protein L36a | 100859914 | 0.006 | 0.33 | 8.36 |
| *SHFM1* | Split hand/foot malformation (ectrodactyly) type 1 | 772237 | 0.004 | 0.33 | 6.48 |
| *C8orf59* | Chromosome 8 open reading frame 59 | 101751649 | 0.013 | 0.34 | 5.75 |
| *PGLYRP2* | Peptidoglycan recognition protein 2 | 693263 | 0.045 | 0.34 | 3.22 |
| *PPIL1* | Peptidylprolyl isomerase like 1 | 100859332 | 0.005 | 0.34 | 4.35 |
| *RPL35A* | Ribosomal protein L35a | 424924 | 0.008 | 0.34 | 8.68 |
| *RPS27A* | Ribosomal protein S27a | 395796 | 0.004 | 0.34 | 8.84 |
| *POLR3K* | RNA polymerase III subunit K | 768505 | 0.012 | 0.34 | 4.41 |
| *HSP90AB1* | Heat shock protein 90 alpha family class B member 1 | 396188 | 0.026 | 0.35 | 6.79 |
| *MRPS16* | Mitochondrial ribosomal protein S16 | 769996 | 0.004 | 0.35 | 5.42 |
| *NDUFA5* | NADH:ubiquinone oxidoreductase subunit A5 | 417753 | 0.005 | 0.35 | 6.53 |
| *POP5* | POP5 homolog, ribonuclease P/MRP subunit | 416973 | 0.012 | 0.35 | 3.96 |
| *RPL38* | Ribosomal protein L38 | 771528 | 0.009 | 0.35 | 8.11 |
| *ANGPT1L* | Angiopoietin-related protein 1-like | 395773 | 0.045 | 0.36 | 10.57 |
| *ATP5I* | ATP synthase, H+ transporting, mitochondrial Fo complex subunit E | 769146 | 0.014 | 0.36 | 7.56 |
| *DPCD* | Deleted in primary ciliary dyskinesia homolog (mouse) | 423852 | 0.004 | 0.36 | 3.2 |
| *DNA2* | DNA replication helicase/nuclease 2 | 423688 | 0.008 | 0.36 | 3.88 |
| *GINS1* | GINS complex subunit 1 | 425297 | 0.041 | 0.36 | 4.02 |
| *NDUFB2* | NADH:ubiquinone oxidoreductase subunit B2 | 418118 | 0.006 | 0.36 | 6.82 |
| *RPL23* | Ribosomal protein L23 | 420001 | 0.009 | 0.36 | 8.98 |
| *H2AZ2* | H2A.Z variant histone 2 | 426617 | 0.006 | 0.37 | 6.88 |
| *S100A6* | S100 calcium binding protein A6 | 373951 | 0.039 | 0.37 | 8.03 |
| *SNRPGP15* | Small nuclear ribonucleoprotein polypeptide G pseudogene 15 | 771344 | 0.004 | 0.37 | 6.41 |
| *TIMM10* | Translocase of inner mitochondrial membrane 10 | 425202 | 0.018 | 0.37 | 3.87 |
| *COA3* | Cytochrome c oxidase assembly factor 3 | 772251 | 0.005 | 0.38 | 4.42 |
| *PTMA* | Prothymosin, alpha | 424931 | 0.007 | 0.38 | 9.05 |
| *RPS27* | Ribosomal protein S27 | 107055140 | 0.014 | 0.38 | 7.71 |
| *SNRPD1* | Small nuclear ribonucleoprotein D1 polypeptide | 776411 | 0.004 | 0.38 | 6 |
| *TIMM13* | Translocase of inner mitochondrial membrane 13 | 100859352 | 0.008 | 0.38 | 4.44 |
| *LOC112533358* | Coiled-coil domain-containing glutamate-rich protein 1-like | 112533358 | 0.024 | 0.4 | 5.11 |
| *COX5B* | Cytochrome c oxidase subunit 5B | 107049545 | 0.008 | 0.4 | 7.1 |
| *NDUFAF8* | NADH:ubiquinone oxidoreductase complex assembly factor 8 | 422071 | 0.014 | 0.4 | 3.01 |
| *RPLP1* | Ribosomal protein lateral stalk subunit P1 | 396262 | 0.004 | 0.4 | 9.11 |
| *SNRPF* | Small nuclear ribonucleoprotein polypeptide F | 417916 | 0.005 | 0.4 | 5.11 |
| *C22orf39* | Chromosome 22 open reading frame 39 | 101751757 | 0.008 | 0.41 | 3.9 |
| *SHMT1* | Serine hydroxymethyltransferase 1 | 416520 | 0.005 | 0.41 | 3.63 |
| *SLIRP* | SRA stem-loop interacting RNA binding protein | 772054 | 0.004 | 0.41 | 5.91 |
| *ANKRD39* | Ankyrin repeat domain 39 | 107057371 | 0.02 | 0.42 | 3.6 |
| *CHTF8* | Chromosome transmission fidelity factor 8 | 769609 | 0.012 | 0.42 | 3.03 |
| *IMP3* | IMP3, U3 small nucleolar ribonucleoprotein, homolog (yeast) | 107053172 | 0.012 | 0.42 | 3.8 |
| *NPB* | Neuropeptide B | 769277 | 0.005 | 0.42 | 3.58 |
| *PPP1R1B* | Protein phosphatase 1 regulatory inhibitor subunit 1B | 426639 | 0.028 | 0.42 | 6.44 |
| *UPB1* | Beta-ureidopropionase 1 | 416949 | 0.037 | 0.43 | 2.71 |
| *CKS1B* | CDC28 protein kinase regulatory subunit 1B | 100134845 | 0.025 | 0.43 | 5.73 |
| *GPX2* | Glutathione peroxidase 2 | 100857454 | 0.02 | 0.43 | 6.44 |
| *MRPS12* | Mitochondrial ribosomal protein S12 | 770472 | 0.005 | 0.43 | 4.41 |
| *COX14* | COX14, cytochrome c oxidase assembly factor | 426880 | 0.015 | 0.44 | 3.77 |
| *CDK2AP2* | Cyclin dependent kinase 2 associated protein 2 | 107053360 | 0.01 | 0.44 | 4.74 |
| *MEA1* | Male-enhanced antigen 1 | 107052849 | 0.028 | 0.44 | 5.59 |
| *NUDT1* | Nudix hydrolase 1 | 416467 | 0.019 | 0.44 | 4.6 |
| *F8A3* | Coagulation factor VIII-associated 3 | 776915 | 0.008 | 0.46 | 3.37 |
| *MNR2* | Homeodomain protein | 395769 | 0.011 | 0.47 | 3.72 |
| *FMC1* | Formation of mitochondrial complex V assembly factor 1 homolog | 107053781 | 0.005 | 0.48 | 3.29 |
| *PCLAF* | PCNA clamp associated factor | 101748248 | 0.012 | 0.5 | 4.05 |
| *COX8A* | Cytochrome c oxidase subunit 8A | 775974 | 0.009 | 0.51 | 6.01 |
| *RRS1* | Ribosome biogenesis regulator homolog | 107052739 | 0.006 | 0.53 | 4.31 |
| *PIGBOS1* | PIGB opposite strand 1 | 105694155 | 0.004 | 0.54 | 4.17 |
| *POLD4* | DNA polymerase delta 4, accessory subunit | 112532567 | 0.031 | 0.57 | 4.43 |
| *GNG11* | G protein subunit gamma 11 | 771999 | 0.009 | 0.57 | 3.17 |
| *LOC107054855* | Corticoliberin-like | 107054855 | 0.008 | 0.62 | 1.88 |
| *NCBP2AS2* | NCBP2 antisense 2 (head to head) | 121111542 | 0.05 | 0.62 | 1.93 |
| *LOC101747821* | Uncharacterized LOC101747821 | 101747821 | 0.027 | 0.73 | 2.03 |
| *IRX6* | Iroquois homeobox 6 | 107054265 | 0.022 | 1.11 | 0.14 |
| *SLC7A13* | Solute carrier family 7 member 13 | 420204 | 0.013 | 2.42 | -0.26 |
